# Supplementary material for: Integrated Taxonomy and Species Diversity of the Historical Chondrichthyan Collection of the Zoology Museum “Pietro Doderlein” at the University of Palermo (Italy)
Source: Biology (Basel). 2025 Aug 26;14(9):1129. doi: 10.3390/biology14091129 (PMC12467001; doi:10.3390/biology14091129)
Supplement: Supplementary file 1 [file biology-14-01129-s001.zip › Appendix.pdf]

Appendix A

Appendix A.1. Catalogue of Chondrichthyan Specimens. The 342 specimens are organized according to the current systematic classification of Chondrichthyes and listed in alphabetical order and following the modern catalogue number.

Class Holocephali
Order Chimaeriformes
Family Chimaeridae Rafinesque, 1815
Genus *Chimaera* Linnaeus, 1758
Species *Chimaera monstrosa* Linnaeus, 1758

• AN45
Present in the historical collection, recovered in Palermo between 1863-1880, stored at the Museum of Zoology "P. Doderlein". Historical inventory number 138.
Skeleton on pedestal.
Adult, total length 55.2 cm.
• AN47
Present in the historical collection, recovered in Palermo between 1863-1880, stored at the Museum of Zoology "P. Doderlein". Historical inventory number 138.
Skeleton on pedestal.
Adult, total length 53.5 cm.
• AN635
Present in the historical collection, recovered in Palermo between 1863-1880, stored at the Museum of Zoology "P. Doderlein". Historical inventory number 151.
Digestive system on pedestal. Currently in poor condition, thoroughly damaged.
Length 18.4 cm.
• PL273
Present in the historical collection, bought in Palermo on the 30.04.1883 by Eredi di Paola, stored at the Museum of Zoology "P. Doderlein". Historical inventory number 594.
Specimen preserved in alcohol.
♀, sub-adult, Total length 43.3 cm.
• P550
Present in the historical collection, bought in Palermo on the 11.06.1886 by Antonio Puleo, stored at the Museum of Zoology "P. Doderlein”. Historical inventory number 803.
Taxidermied specimen on pedestal. Currently in mediocre condition, damage in some points.
♀, adult, Total length 55.4 cm.
• P555
Present in the historical collection, bought in Palermo on the 11.06.1886 by Antonio Puleo, stored at the Museum of Zoology "P. Doderlein”. Historical inventory number 803.
Taxidermied specimen on pedestal.
♂, adult, Total length 53.2 cm.
• P584
Present in the historical collection, recovered in Palermo between 1863-1880, stored at the Museum of Zoology "P. Doderlein". Historical inventory number 118.
Taxidermied specimen on pedestal.
♀, adult, Total length 54.5 cm.

Class Elasmobranchii
Superorder Batoidea
Order Rajiformes
Family Rajidae de Blainville, 1816
Unidentified taxa

• AN81
Present in the historical collection, recovered in Palermo on an unknown date, stored at the Museum of Zoology "P. Doderlein". Historical inventory number 2650 bis.
Jaw on pedestal.
Length 17.4 cm.
• AN121
Present in the historical collection, recovered in Palermo on an unknown date, stored at the Museum of Zoology "P. Doderlein". Historical inventory number 2650 bis.
Jaw on pedestal.
♂, length 22.5 cm.
• PL360
Present in the historical collection, recovered in Palermo on an unknown date, stored at the Museum of Zoology "P. Doderlein". Historical inventory number unknown.
Specimen preserved in alcohol.
♀, total length 19.7 cm.
• P586
Present in the historical collection, recovered in Palermo between 1863-1880, stored at the Museum of Zoology "P. Doderlein". Historical inventory number 121.
Taxidermied specimen on pedestal.
♀, total length 25.8 cm.
• P628
Present in the historical collection, recovered in Palermo between 1863-1880, stored at the Museum of Zoology "P. Doderlein". Historical inventory number 121.
Taxidermied specimens on pedestal.
♀, total length 46.3 cm.

Genus *Dipturus* Rafinesque, 1810
Species *Dipturus batis* Linnaeus, 1758

• AN39
Present in the historical collection, recovered in Palermo between 1863-1880, stored at the Museum of Zoology "P. Doderlein". Historical inventory number 143.
Skeleton on pedestal.
Total length 39.4 cm.
• AN657
Present in the historical collection, recovered in Palermo between 1863-1880, stored at the Museum of Zoology "P. Doderlein". Historical inventory number 151.
Digestive system on pedestal. Currently in poor condition, broken in several parts.
Length 39.7 cm.
• P653
Present in the historical collection, recovered in Palermo on an unknown date, stored at the Museum of Zoology "P. Doderlein". Historical inventory number unknown.
Taxidermied specimen on pedestal.
♀, sub-adult, Total length 120.6 cm.
• P660
Present in the historical collection, recovered in Palermo on an unknown date, stored at the Museum of Zoology "P. Doderlein". Historical inventory number unknown.
Taxidermied specimen on pedestal.
♀, adult, Total length 214.6 cm.

Species *Dipturus nidarosiensis* Storm, 1881

• P651
Present in the historical collection, recovered in Palermo on an unknown date, stored at the Museum of Zoology "P. Doderlein". Historical inventory number unknown.
Taxidermied specimen on pedestal.
♀, sub-adult, Total length 145.4 cm.

Species *Dipturus oxyrinchus* Linnaeus, 1758

• AN67
Present in the historical collection, recovered in Palermo on an unknown date, stored at the Museum of Zoology "P. Doderlein". Historical inventory number 2650 bis.
Jaw on pedestal.
♀, length 9.8 cm.
• AN104
Present in the historical collection, recovered in Palermo between 1863-1880, stored at the Museum of Zoology "P. Doderlein". Historical inventory number 143.
Skeleton on pedestal.
Total length 26.5 cm.
• AN634
Present in the historical collection, recovered in Palermo between 1863-1880, stored at the Museum of Zoology "P. Doderlein". Historical inventory number 151.
Digestive system on pedestal. Currently in poor condition, broken in several parts.
Length 18.8 cm.
• AN664
Present in the historical collection, recovered in Palermo between 1863-1880, stored at the Museum of Zoology "P. Doderlein". Historical inventory number 139.
Skeleton on pedestal. Currently in poor condition, broken pectoral fins.
♀, sub-adult, total length 78.2 cm.
• P568
Present in the historical collection, recovered in Palermo between 1863-1880, stored at the Museum of Zoology "P. Doderlein". Historical inventory number 120.
Taxidermied specimen on pedestal.
♂, sub- adult, total length 90 cm.
• P572
Present in the historical collection, recovered in Palermo between 1863-1880, stored at the Museum of Zoology "P. Doderlein". Historical inventory number 120.
Taxidermied specimen on pedestal.
♀, adult, total length 102 cm.
• P621
Present in the historical collection, recovered in Palermo between 1863-1880, stored at the Museum of Zoology "P. Doderlein". Historical inventory number 121.
Taxidermied specimen on pedestal. Currently in poor condition, slightly raised taxidermy.
♂, sub- adult, total length 64 cm.

Genus *Leucoraja* Malm, 1877
Species *Leucoraja circularis* Couch, 1838

• AN8
Present in the historical collection, recovered in Palermo between 1863-1880, stored at the Museum of Zoology "P. Doderlein". Historical inventory number 147.
Jaw on pedestal.
Length 9.7 cm.

• AN13
Present in the historical collection, recovered in Palermo between 1863-1880, stored at the Museum of Zoology "P. Doderlein". Historical inventory number 139.
Skeleton on pedestal. Currently in poor condition, the tail is missing.
♂, total length 46.6 cm.
• AN658
Present in the historical collection, recovered in Palermo between 1863-1880, stored at the Museum of Zoology "P. Doderlein". Historical inventory number 151.
Digestive system on pedestal.
Length 16.2 cm.
• P566
Present in the historical collection, recovered in Palermo between 1863-1880, stored at the Museum of Zoology "P. Doderlein". Historical inventory number 121.
Taxidermied specimen on pedestal.
♂, sub- adult, total length 47.1 cm.
• P569
Present in the historical collection, recovered in Palermo between 1863-1880, stored at the Museum of Zoology "P. Doderlein". Historical inventory number 120.
Taxidermied specimen on pedestal.
♀, adult, total length 92.5 cm.
• P605
Present in the historical collection, recovered in Palermo between 1863-1880, stored at the Museum of Zoology "P. Doderlein". Historical inventory number 121.
Taxidermied specimen on pedestal.
♂, sub-adult, total length 56 cm.
• P609
Present in the historical collection, bought in Palermo on the 31.07.1885 by Eredi di Paola, stored at the Museum of Zoology "P. Doderlein". Historical inventory number 762.
Taxidermied specimen on pedestal.
♀, sub- adult, total length 36.9 cm.
• P627
Present in the historical collection, recovered in Palermo between 1863-1880, stored at the Museum of Zoology "P. Doderlein". Historical inventory number 121.
Taxidermied specimen on pedestal.
♀, sub- adult, total length 57 cm.

Species *Leucoraja fullonica* Linnaeus, 1758

• P577
Present in the historical collection, bought in Palermo on the 16.12.1884 by Eredi di Paola, stored at the Museum of Zoology "P. Doderlein". Historical inventory number 716.
Taxidermied specimen on pedestal.
♀, sub- adult, total length 78 cm.

Species *Leucoraja naevus* Müller & Henle, 1841

• AN9
Present in the historical collection, recovered in Palermo between 1863-1880, stored at the Museum of Zoology "P. Doderlein". Historical inventory number 139.
Skeleton on pedestal. Currently in poor condition, the tail is missing.
♂, total length 26.6 cm.
• AN10

Present in the historical collection, recovered in Palermo between 1863-1880, stored at the Museum of Zoology "P. Doderlein". Historical inventory number 139.
Skeleton on pedestal. Currently in poor condition, the tail is missing.
♀, total length 23.8 cm.
• P565
Present in the historical collection, recovered in Palermo between 1863-1880, stored at the Museum of Zoology "P. Doderlein". Historical inventory number 121.
Taxidermied specimen on pedestal.
♂, sub-adult, total length 39 cm.

Genus *Raja* Linnaeus, 1758
Species *Raja asterias* Delaroche, 1809

• AN1
Present in the historical collection, recovered in Palermo between 1863-1880, stored at the Museum of Zoology "P. Doderlein". Historical inventory number 139.
Skeleton on pedestal.
♂, sub- adult, total length 54 cm.
• AN4
Present in the historical collection, recovered in Palermo between 1863-1880, stored at the Museum of Zoology "P. Doderlein". Historical inventory number 147.
Jaw on pedestal.
Length 9.5 cm.
• AN5
Present in the historical collection, recovered in Palermo between 1863-1880, stored at the Museum of Zoology "P. Doderlein". Historical inventory number 139.
Skeleton on pedestal.
♀, sub- adult, total length 55 cm.
• AN11
Present in the historical collection, bought in Palermo on 30.04.1882 by Eredi di Paola, stored at the Museum of Zoology "P. Doderlein". Historical inventory number 486.
Jaw on pedestal.
Length 9.5 cm.
• AN580
Present in the historical collection, recovered in Palermo between 1863-1880, stored at the Museum of Zoology "P. Doderlein". Historical inventory number 151.
Digestive system on pedestal. Currently in poor condition, thoroughly damaged.
Total length 13.2 cm.
• P570
Present in the historical collection, recovered in Palermo between 1863-1880, stored at the Museum of Zoology "P. Doderlein". Historical inventory number 121.
Taxidermied specimens on pedestal.
♂, ♀, juveniles, total length 62 cm.
• P607
Present in the historical collection, bought in Palermo on the 31.12.1882 by Eredi di Paola, stored at the Museum of Zoology "P. Doderlein". Historical inventory number 567.
Taxidermied specimens on pedestal.
♀, juveniles, total length 49.2 cm.
• P610
Present in the historical collection, recovered in Palermo between 1863-1880 stored at the Museum of Zoology "P. Doderlein". Historical inventory number 121.
Taxidermied specimens on pedestal.

♀, sub-adult, total length 61 cm.
• P624
Present in the historical collection, bought in Palermo on the 16.08.1884 by Eredi di Paola, stored at the Museum of Zoology "P. Doderlein". Historical inventory number 695.
Taxidermied specimens on pedestal.
♂, sub-adult, total length 44.5 cm.

Species *Raja brachyura* Lafont, 1871

• AN35
Present in the historical collection, recovered in Palermo between 1863-1880, stored at the Museum of Zoology "P. Doderlein". Historical inventory number 139.
Skeleton on pedestal. Currently in poor condition, broken in several parts.
♀, adult, total length 85 cm.
• AN51
Present in the historical collection, recovered in Palermo on an unknown date stored at the Museum of Zoology "P. Doderlein". Historical inventory number 2650 bis.
Jaw on pedestal.
Length 10.9 cm.
• AN69
Present in the historical collection, recovered in Palermo on an unknown date, stored at the Museum of Zoology "P. Doderlein". Historical inventory number 2650 bis.
Jaw on pedestal.
Length 9.5 cm.
• AN630
Present in the historical collection, recovered in Palermo between 1863-1880, stored at the Museum of Zoology "P. Doderlein". Historical inventory number 151.
Digestive system on pedestal. Currently in poor condition, thoroughly damaged
Length 11.9 cm.
• P593
Present in the historical collection, recovered in Palermo between 1863-1880, stored at the Museum of Zoology "P. Doderlein". Historical inventory number 120.
Taxidermied specimens on pedestal.
♂, adult, total length 84 cm.
• P618
Present in the historical collection, recovered in Palermo between 1863-1880, stored at the Museum of Zoology "P. Doderlein". Historical inventory number 120.
Taxidermied specimens on pedestal.
♀, adult, total length 79 cm.
• P645
Present in the historical collection, recovered in Palermo between 1863-1880, stored at the Museum of Zoology "P. Doderlein". Historical inventory number 120.
Taxidermied specimens on pedestal.
♂, adult, total length 83 cm.

Species *Raja clavata* Linnaeus, 1758

• AN29
Present in the historical collection, recovered in Palermo between 1863-1880, stored at the Museum of Zoology "P. Doderlein". Historical inventory number 139.
Skeleton on pedestal. Currently in poor condition, with part of the tail missing.
♀, adult, total length 66 cm.

• AN30
Present in the historical collection, recovered in Palermo between 1863-1880, stored at the Museum of Zoology "P. Doderlein". Historical inventory number 139.
Skeleton on pedestal.
♂, adult, total length 60 cm.
• AN99
Present in the historical collection, recovered in Palermo on an unknown date, stored at the Museum of Zoology "P. Doderlein". Historical inventory number 2650 bis.
Jaw on pedestal.
Length 10.4 cm.
• AN103
Present in the historical collection, recovered in Palermo on an unknown date, stored at the Museum of Zoology "P. Doderlein". Historical inventory number 2650 bis.
Jaw on pedestal.
♀, length 9.9 cm.
• AN120
Present in the historical collection, recovered in Palermo on an unknown date, stored at the Museum of Zoology "P. Doderlein". Historical inventory number 2650 bis.
Jaw on pedestal.
Length 10.1 cm.
• AN568
Present in the historical collection, recovered in Palermo between 1863-1880, stored at the Museum of Zoology "P. Doderlein". Historical inventory number 151.
Digestive system on pedestal. Currently in poor condition, thoroughly damaged.
Length 10.2 cm.
• P594
Present in the historical collection, recovered in Palermo between 1863-1880, stored at the Museum of Zoology "P. Doderlein". Historical inventory number 120.
Taxidermied specimens on pedestal.
♀, adult, total length 78.5 cm.
• P611
Present in the historical collection, recovered in Palermo between 1863-1880, stored at the Museum of Zoology "P. Doderlein". Historical inventory number 121.
Taxidermied specimens on pedestal.
♂, sub-adult, total length 58 cm.
• P617
Present in the historical collection, recovered in Palermo between 1863-1880 stored at the Museum of Zoology "P. Doderlein". Historical inventory number 121.
Taxidermied specimens on pedestal.
♂, sub-adult, total length 64 cm.
• P620
Present in the historical collection, recovered in Palermo between 1863-1880 stored at the Museum of Zoology "P. Doderlein". Historical inventory number 121.
Taxidermied specimens on pedestal.
♀, sub-adult, total length 41.2 cm.
• P623
Present in the historical collection, recovered in Palermo between 1863-1880, stored at the Museum of Zoology "P. Doderlein". Historical inventory number 121.
Taxidermied specimens on pedestal.
♂, adult, total length 70 cm.

Species *Raja miraletus* Linnaeus, 1758

• AN2
Present in the historical collection, recovered in Palermo between 1863-1880, stored at the Museum of Zoology "P. Doderlein". Historical inventory number 139.
Skeleton on pedestal.
♂, adult, total length 43.1 cm.
• AN3
Present in the historical collection, recovered in Palermo on the 30.04.1882 by Eredi di Paola, stored at the Museum of Zoology "P. Doderlein". Historical inventory number 489.
Jaw on pedestal.
♀, length 9.5 cm.
• AN6
Present in the historical collection, recovered in Palermo between 1863-1880, stored at the Museum of Zoology "P. Doderlein". Historical inventory number 139.
Skeleton on pedestal.
♀, sub-adult, total length 35.8 cm.
• AN582
Present in the historical collection, recovered in Palermo on an unknown date, stored at the Museum of Zoology "P. Doderlein". Historical inventory number unknown.
Digestive system on pedestal. Currently in poor condition, thoroughly damaged.
Length 9.2 cm.
• P567
Present in the historical collection, recovered in Palermo between 1863-1880, stored at the Museum of Zoology "P. Doderlein". Historical inventory number 121.
Taxidermied specimens on pedestal.
♂, adult, total length 44.9 cm.
• P613
Present in the historical collection, recovered in Palermo between 1863-1880, stored at the Museum of Zoology "P. Doderlein". Historical inventory number 121.
Taxidermied specimens on pedestal.
♀, sub-adult, total length 38.2 cm.
• P615
Present in the historical collection, recovered in Palermo between 1863-1880, stored at the Museum of Zoology "P. Doderlein". Historical inventory number 121.
Taxidermied specimens on pedestal. Currently in poor condition, broken in several parts.
♀, adult, total length 45.3 cm.

Species *Raja polystigma* Regan, 1923

• P602
Present in the historical collection, recovered in Palermo between 1863-1880, stored at the Museum of Zoology "P. Doderlein". Historical inventory number 121.
Taxidermied specimen on pedestal.
♂, sub-adult, total length 36.9 cm.
• P604
Present in the historical collection, recovered in Palermo between 1863-1880, stored at the Museum of Zoology "P. Doderlein". Historical inventory number 121.
Taxidermied specimen on pedestal.
♀, juvenil, total length 31 cm.
• P612
Present in the historical collection, bought in Palermo on the 31.12.1886 by Antonio Puleo, stored at the Museum of Zoology "P. Doderlein”. Historical inventory number 847.

Taxidermied specimen on pedestal.
♂, adult, total length 57.3 cm.
• P622
Present in the historical collection, bought in Palermo on the 05.04.1884 by Eredi di Paola, stored at the Museum of Zoology "P. Doderlein". Historical inventory number 658.
Taxidermied specimen on pedestal.
♀, adult, total length 50.2 cm.

Species *Raja radula* Delaroche, 1809

• AN20
Present in the historical collection, recovered in Palermo between 1863-1880, stored at the Museum of Zoology "P. Doderlein". Historical inventory number 139.
Skeleton on pedestal. Currently in poor condition, missing tail.
♂, sub-adult, total length 38.5 cm.
• AN21
Present in the historical collection, recovered in Palermo between 1863-1880, stored at the Museum of Zoology "P. Doderlein". Historical inventory number 139.
Skeleton on pedestal. Currently in poor condition, damaged.
♀, sub-adult, total length 38.8 cm.
• AN22
Present in the historical collection, recovered in Palermo between 1863-1880, stored at the Museum of Zoology "P. Doderlein". Historical inventory number 139.
Skeleton on pedestal. Currently in poor condition, damaged.
♂, sub-adult, total length 40.5 cm.
• AN23
Present in the historical collection, recovered in Palermo between 1863-1880, stored at the Museum of Zoology "P. Doderlein". Historical inventory number 139.
Skeleton on pedestal. Currently in poor condition, missing tail.
Total length 34.3 cm.
• AN58
Present in the historical collection, bought in Palermo on the 30.04.1882 by Eredi di Paola, stored at the Museum of Zoology "P. Doderlein". Historical inventory number 494.
Jaw on pedestal.
♂, length 6.3 cm.
• AN96
Present in the historical collection, recovered in Palermo on an unknown date, stored at the Museum of Zoology "P. Doderlein". Historical inventory number 2650 bis.
Jaw on pedestal.
Length 9.6 cm.
• P597
Present in the historical collection, recovered in Palermo between 1863-1880, stored at the Museum of Zoology "P. Doderlein". Historical inventory number 121.
Taxidermied specimen on pedestal.
♂, adult, Total length 49.4 cm.
• P606
Present in the historical collection, bought in Palermo on the 31.03.1885 by Eredi di Paola, stored at the Museum of Zoology "P. Doderlein". Historical inventory number 741.
Taxidermied specimen on pedestal.
♂, total length 46.1 cm.
• P608

Present in the historical collection, recovered in Palermo between 1863-1880, stored at the Museum of Zoology "P. Doderlein". Historical inventory number 121.
Taxidermied specimen on pedestal.
♀, sub-adult, Total length 42.5 cm.
• P616
Present in the historical collection, recovered in Palermo between 1863-1880, stored at the Museum of Zoology "P. Doderlein". Historical inventory number 121.
Taxidermied specimen on pedestal. Currently in poor condition, the upper fin is broken.
♂, sub-adult, total length 39.5 cm.
• P619
Present in the historical collection, bought in Palermo on the 31.03.1885 by Eredi di Paola, stored at the Museum of Zoology "P. Doderlein". Historical inventory number 737 bis.
Taxidermied specimen on pedestal.
♀, adult, total length 48.2 cm.
• P625
Present in the historical collection, recovered in Palermo between 1863-1880, stored at the Museum of Zoology "P. Doderlein". Historical inventory number 121.
Taxidermied specimen on pedestal.
♀, adult, Total length 53.6 cm.

Genus *Rostroraja* Hulley, 1972
Species *Rostroraja alba* Lacepède, 1803

• AN7
Present in the historical collection, recovered in Palermo between 1863-1880, stored at the Museum of Zoology "P. Doderlein". Historical inventory number 139.
Skeleton on pedestal. Currently in poor condition, broken in several parts.
♀, total length 43.1 cm.
• AN12
Present in the historical collection, recovered in Palermo on an unknown date, stored at the Museum of Zoology "P. Doderlein". Historical inventory number 2650 bis.
Jaw on pedestal.
Length 12.6 cm.
• AN34
Present in the historical collection, recovered in Palermo on an unknown date, stored at the Museum of Zoology "P. Doderlein". Historical inventory number 2648 bis.
Skeleton on pedestal.
♀, juvenile, total length 69.5 cm.
• AN56
Present in the historical collection, recovered in Palermo between 1863-1880, stored at the Museum of Zoology "P. Doderlein". Historical inventory number 147.
Skeleton on pedestal.
Total length 20.6 cm.
• AN74
Present in the historical collection, recovered in Palermo on an unknown date, stored at the Museum of Zoology "P. Doderlein". Historical inventory number 2650 bis.
Jaw on pedestal.
♀, Length 14.4 cm.
• AN87
Present in the historical collection, recovered in Palermo between 1863-1880, stored at the Museum of Zoology "P. Doderlein". Historical inventory number 147.
Jaw on pedestal.

Length 18.2 cm.
• AN92
Present in the historical collection, recovered in Palermo on an unknown date, stored at the Museum of Zoology "P. Doderlein". Historical inventory number 2650 bis.
Jaw on pedestal.
Length 6.4 cm.
• AN149
Present in the historical collection, recovered in Palermo on an unknown date, stored at the Museum of Zoology "P. Doderlein". Historical inventory number 2650 bis.
Jaw on pedestal.
Length 16.9 cm.
• AN406
Present in the historical collection, recovered in Palermo on an unknown date, stored at the Museum of Zoology "P. Doderlein". Historical inventory number 2650 bis.
Jaw on pedestal.
Length 20.3 cm.
• AN487
Present in the historical collection, recovered in Palermo between 1863-1880, stored at the Museum of Zoology "P. Doderlein". Historical inventory number 153.
Branchial and circulatory system on a pedestal.
Length 12.5 cm.
• AN631
Present in the historical collection, recovered in Palermo on an unknown date, stored at the Museum of Zoology "P. Doderlein". Historical inventory number unknown.
Oviduct on a pedestal. Currently in poor condition, broken in several parts.
Length 23.4 cm.
• AN654
Present in the historical collection, recovered in Palermo between 1863-1880, stored at the Museum of Zoology "P. Doderlein". Historical inventory number 151.
Digestive system on pedestal. Currently in poor condition, thoroughly damaged.
Length 35.5 cm.
• P574
Present in the historical collection, recovered in Palermo between 1863-1880, stored at the Museum of Zoology "P. Doderlein". Historical inventory number 120.
Taxidermied specimen on pedestal.
♀, juvenile, total length 86.9 cm.
• P583
Present in the historical collection, recovered in Palermo on an unknown date, stored at the Museum of Zoology "P. Doderlein". Historical inventory number unknown.
Taxidermied specimen on pedestal.
♀, sub-adult, total length 130 cm.
• P588
Present in the historical collection, recovered in Palermo between 1863-1880, stored at the Museum of Zoology "P. Doderlein". Historical inventory number 121.
Taxidermied specimen on pedestal.
♂, juvenile, total length 42.7 cm.
• P592
Present in the historical collection, bought in Palermo on the 31.03.1885 by Eredi di Paola, stored at the Museum of Zoology "P. Doderlein". Historical inventory number 742.
Taxidermied specimen on pedestal.
♀, juvenile, total length 63.3 cm.
• P652

Present in the historical collection, recovered in Palermo on an unknown date, stored at the Museum of Zoology "P. Doderlein". Historical inventory number unknown.
Taxidermied specimen on pedestal.
♀, sub-adult, total length 112 cm.

Order Myliobatiformes
Family Dasyatidae Jordan & Gilbert, 1879
Genus *Bathytoshia* Whitley, 1933
Species *Bathytoshia centroura* Mitchill, 1815

- AN62

Present in the historical collection, recovered in Palermo on an unknown date, stored at the Museum of Zoology "P. Doderlein". Historical inventory number 2650 bis.
Jaw on pedestal.
♂, length 16.2 cm.

- AN76

Present in the historical collection, recovered in Palermo on an unknown date, stored at the Museum of Zoology "P. Doderlein". Historical inventory number 2648 bis.
Skeleton on pedestal.
Adult, total length 221.7 cm.

- AN78

Present in the historical collection, recovered in Palermo on an unknown date, stored at the Museum of Zoology "P. Doderlein". Historical inventory number 2650 bis.
Jaw on pedestal.
Length 27.1 cm.

- AN116

Present in the historical collection, recovered in Palermo on an unknown date, stored at the Museum of Zoology "P. Doderlein". Historical inventory number 2650 bis.
Jaw on pedestal.
♀, length 18.9 cm.

- AN127

Present in the historical collection, recovered in Palermo on an unknown date, stored at the Museum of Zoology "P. Doderlein". Historical inventory number 2650 bis.
Jaw on pedestal.
Length 19.8 cm.

- AN138

Present in the historical collection, recovered in Palermo on an unknown date, stored at the Museum of Zoology "P. Doderlein". Historical inventory number 2650 bis.
Jaw on pedestal.
Length 21.7 cm.

- AN620

Present in the historical collection, recovered in Palermo between 1863-1880, stored at the Museum of Zoology "P. Doderlein". Historical inventory number 151.
Digestive system on pedestal.
Length 52.1 cm.

- P576

Present in the historical collection, recovered in Palermo between 1863-1880, stored at the Museum of Zoology "P. Doderlein". Historical inventory number 121.
Taxidermied specimen on pedestal.
♀, juvenile, Total length 25.2 cm.

- P600

Present in the historical collection, bought in Palermo on the 19.12.82 by Nunzio Tagliavia, stored at the Museum of Zoology "P. Doderlein". Historical inventory number 551.
Taxidermized tail.
Total length 198.5 cm.

Genus *Dasyatis* Rafinesque, 1810
Species *Dasyatis pastinaca* Linnaeus, 1758

- AN24

Present in the historical collection, recovered in Palermo between 1863-1880, stored at the Museum of Zoology "P. Doderlein". Historical inventory number 139.
Skeleton on pedestal. Currently in poor condition, broken in several parts.
♂, juvenile, total length 44.7 cm.

- AN71

Present in the historical collection, recovered in Palermo on an unknown date, stored at the Museum of Zoology "P. Doderlein". Historical inventory number 2650 bis.
Jaw on pedestal.
♀, length 11.2 cm.

- AN107

Present in the historical collection, recovered in Palermo on an unknown date, stored at the Museum of Zoology "P. Doderlein". Historical inventory number 2650 bis.
Jaw on pedestal.
Length 6.9 cm.

- AN530

Present in the historical collection, recovered in Palermo between 1863-1880, stored at the Museum of Zoology "P. Doderlein". Historical inventory number 151.
Digestive system on pedestal. Currently in poor condition, broken in several parts.
Length 9.9 cm.

- AN537

Present in the historical collection, recovered in Palermo between 1863-1880, stored at the Museum of Zoology "P. Doderlein". Historical inventory number 151.
Digestive system on pedestal. Currently in poor condition, broken in several parts.
Length 20.2 cm.

- AN589

Present in the historical collection, recovered in Palermo between 1863-1880, stored at the Museum of Zoology "P. Doderlein". Historical inventory number 151.
Digestive system on pedestal.
Length 27.7 cm

- AN648

Present in the historical collection, recovered in Palermo between 1863-1880, stored at the Museum of Zoology "P. Doderlein". Historical inventory number 151.
Digestive system on pedestal.
Length 25.5cm.

- P579

Present in the historical collection, recovered in Palermo between 1863-1880, stored at the Museum of Zoology "P. Doderlein". Historical inventory number 120.
Taxidermied specimen on pedestal.
♀, juvenile, total length 76.3 cm.

- P614

Present in the historical collection, recovered in Palermo between 1863-1880, stored at the Museum of Zoology "P. Doderlein". Historical inventory number 121.
Taxidermied specimen on pedestal.

♀, juvenile, total length 32.3 cm.
• P654
Present in the historical collection, bought in Palermo on the 02.12.1884 by Nunzio Tagliavia, stored at the Museum of Zoology "P. Doderlein". Historical inventory number 709.
Taxidermied specimen on pedestal.
♂, sub-adult, Total length 115.5 cm.

Genus *Pteroplatytrygon* Fowler, 1910
Species *Pteroplatytrygon violacea* Bonaparte, 1832

• AN25
Present in the historical collection, recovered in Palermo between 1863-1880, stored at the Museum of Zoology "P. Doderlein". Historical inventory number 139.
Skeleton on pedestal.
♀, sub-adult, total length 59.9 cm.
• AN137
Present in the historical collection, recovered in Palermo between 1863-1880, stored at the Museum of Zoology "P. Doderlein". Historical inventory number 147.
Jaw on pedestal.
Length 6.2 cm.
• PL10
Present in the historical collection, recovered in Palermo on an unknown date, stored at the Museum of Zoology "P. Doderlein". Historical inventory number unknown.
Two specimens preserved in alcohol.
♀, ♂, juveniles, total length 10.5 cm.
• P599
Present in the historical collection, recovered in Palermo between 1863-1880, stored at the Museum of Zoology "P. Doderlein". Historical inventory number 120.
Taxidermied specimen on pedestal. Currently in poor condition, broken in the tail.
♀, sub-adult, total length 65.6 cm.

Family Gymnuridae Fowler, 1934
Genus *Gymnura* van Hasselt, 1823
Species *Gymnura altavela* Linnaeus, 1758

• AN36
Present in the historical collection, recovered in Palermo between 1863-1880, stored at the Museum of Zoology "P. Doderlein". Historical inventory number 139.
Skeleton on pedestal. Currently in poor condition, missing tail.
Total length 24.4 cm.
• AN545
Present in the historical collection, recovered in Palermo on an unknown date, stored at the Museum of Zoology "P. Doderlein". Historical inventory number unknown.
Digestive system on pedestal. Currently in poor condition, thoroughly damaged.
Length 33.5 cm.
• AN611
Present in the historical collection, recovered in Palermo between 1863-1880, stored at the Museum of Zoology "P. Doderlein". Historical inventory number 151.
Digestive system on pedestal.
Length 12.5 cm.
• AN616

Present in the historical collection, recovered in Palermo between 1863-1880, stored at the Museum of Zoology "P. Doderlein". Historical inventory number 151.
Digestive system on pedestal. Currently in poor condition, thoroughly damaged.
Length 14.3 cm.
• P571
Present in the historical collection, recovered in Palermo between 1863-1880, stored at the Museum of Zoology "P. Doderlein". Historical inventory number 121.
Taxidermied specimen on pedestal.
♂, juvenile, total length 41.9 cm.
• P573
Present in the historical collection, recovered in Palermo between 1863-1880, stored at the Museum of Zoology "P. Doderlein". Historical inventory number 120.
Taxidermied specimen on pedestal. Currently in poor condition.
♂, juvenile, total length 44.6 cm.
• P595
Present in the historical collection, bought in Palermo on the 16.12.1884 by Eredi diPaola, stored at the Museum of Zoology "P. Doderlein". Historical inventory number 715.
Taxidermied specimen on pedestal.
♀, juvenile, total length 22.5 cm.

Family Mobulidae Gill, 1893
Genus *Mobula* Rafinesque, 1810
Species *Mobula mobular* Bonnaterre, 1788

• AN26
Present in the historical collection, bought in Palermo on the 31.07.1881 by Eredi diPaola, stored at the Museum of Zoology "P. Doderlein". Historical inventory number 439.
Skull on pedestal.
Length 41.9 cm.
• AN102
Present in the historical collection, recovered in Palermo between 1863-1880, stored at the Museum of Zoology "P. Doderlein". Historical inventory number 147.
Jaw on pedestal.
Length 35.2 cm.
• P630
Present in the historical collection, recovered in Palermo between 1863-1880, stored at the Museum of Zoology "P. Doderlein". Historical inventory number 123.
Taxidermied skull.
Length 80.5 cm.

Family Myliobatidae Bonaparte, 1835
Genus *Aetomylaeus* Garman, 1908
Species *Aetomylaeus bovinus* Geoffroy Saint-Hilaire, 1817

• AN17
Present in the historical collection, recovered in Palermo between 1863-1880, stored at the Museum of Zoology "P. Doderlein". Historical inventory number 139.
Skeleton on pedestal. Currently in poor condition, broken in several parts.
♀, juvenile, total length 25.1 cm.
• AN37
Present in the historical collection, recovered in Palermo between 1863-1880, stored at the Museum of Zoology "P. Doderlein". Historical inventory number 143.

Skull on pedestal.
Length 52.5 cm.
• AN82
Present in the historical collection, recovered in Palermo on an unknown date, stored at the Museum of Zoology "P. Doderlein". Historical inventory number 2650 bis.
Jaw on pedestal.
Length 14.7 cm.
• AN114
Present in the historical collection, recovered in Palermo on an unknown date, stored at the Museum of Zoology "P. Doderlein". Historical inventory number 2650 bis.
Jaw on pedestal.
Length 12.8 cm.
• AN136
Present in the historical collection, recovered in Palermo on an unknown date, stored at the Museum of Zoology "P. Doderlein". Historical inventory number 2650 bis.
Skull on pedestal.
Length 11.2 cm.
• AN539
Present in the historical collection, recovered in Palermo between 1863-1880, stored at the Museum of Zoology "P. Doderlein". Historical inventory number 151.
Digestive system on pedestal. Currently in poor condition, thoroughly damaged.
Length 8.4 cm.
• AN594
Present in the historical collection, recovered in Palermo between 1863-1880, stored at the Museum of Zoology "P. Doderlein". Historical inventory number 151.
Digestive system on pedestal.
Length 16.6 cm.
• PL33
Present in the historical collection, recovered in Palermo on an unknown date, stored at the Museum of Zoology "P. Doderlein". Historical inventory number unknown.
Two specimens preserved in alcohol.
♀, juveniles, total length 13.2 cm.
• P582
Present in the historical collection, recovered in Palermo between 1863-1880, stored at the Museum of Zoology "P. Doderlein". Historical inventory number 120.
Taxidermied specimen on pedestal.
♀, juvenile, total length 61.3 cm.
• P596
Present in the historical collection, recovered in Palermo between 1863-1880, stored at the Museum of Zoology "P. Doderlein". Historical inventory number 119.
Taxidermied specimen on pedestal.
♀, sub-adult, total length 118.9 cm.

Genus *Myliobatis* Cuvier, 1816
Species *Myliobatis aquila* Linnaeus, 1758

• AN16
Present in the historical collection, recovered in Palermo between 1863-1880, stored at the Museum of Zoology "P. Doderlein". Historical inventory number 139.
Skeleton on pedestal. Currently in poor condition, broken in several parts.
♂, juvenile, total length 42.5 cm.
• AN88

Present in the historical collection, recovered in Palermo on an unknown date, stored at the Museum of Zoology "P. Doderlein". Historical inventory number 2650 bis.
Jaw on pedestal.
Length 21.5 cm.
• AN577
Present in the historical collection, recovered in Palermo between 1863-1880, stored at the Museum of Zoology "P. Doderlein". Historical inventory number 151.
Digestive system on pedestal.
Length 26.9 cm.
• AN646
Present in the historical collection, recovered in Palermo between 1863-1880, stored at the Museum of Zoology "P. Doderlein". Historical inventory number 151.
Digestive system on pedestal. Currently in poor condition, broken in two parts.
Length 20.4 cm.
• AN647
Present in the historical collection, recovered in Palermo between 1863-1880, stored at the Museum of Zoology "P. Doderlein". Historical inventory number 151.
Digestive system on pedestal.
Length 38.9 cm.
• P598
Present in the historical collection, recovered in Palermo between 1863-1880, stored at the Museum of Zoology "P. Doderlein". Historical inventory number 121.
Taxidermied specimen on pedestal.
♀, juvenile, total length 84.3 cm.
• P601
Present in the historical collection, recovered in Palermo between 1863-1880, stored at the Museum of Zoology "P. Doderlein". Historical inventory number 121.
Taxidermied specimen on pedestal.
♀, juvenile, total length 37.1 cm.

Order Rhinopristiformes
Family Glaucostegidae Last, Séret & Naylor, 2016
Genus *Glaucostegus* Bonaparte, 1846
Species *Glaucostegus cemiculus* Geoffroy Saint-Hilaire, 1817

• P562
Present in the historical collection, bought in Palermo on the 30.04.1883 by Eredi di Paola, stored at the Museum of Zoology "P. Doderlein”. Historical inventory number 601.
Taxidermied specimen on pedestal.
♂, sub-adult, Total length 118.5 cm.
• P585
Present in the historical collection, bought in Palermo on the 11.06.1886 by Antonio Puleo, stored at the Museum of Zoology "P. Doderlein”. Historical inventory number 804.
Taxidermied specimen on pedestal.
♀, juvenile, Total length 34.5 cm.
• P649
Present in the historical collection, recovered in Palermo on an unknown date, stored at the Museum of Zoology "P. Doderlein". Historical inventory number 2590 bis.
Taxidermied specimen on pedestal.
♀, sub-adult, total length 165.8 cm.
• P650

Present in the historical collection, specimen exchanged on the 03.04.1882 with the Zoological Museum of Vertebrates in Florence by Prof. Pietro Doderlein, stored at the Museum of Zoology "P. Doderlein". Historical inventory number 478.
Taxidermied specimen on pedestal.
♀, adult, total length 182.7 cm.

Family Pristidae Bonaparte, 1835
Genus *Anoxypristis* White & Moy-Thomas, 1941
Species *Anoxypristis cuspidata* Latham, 1794

• AN182
Present in the historical collection, recovered in Palermo between 1863-1880, stored at the Museum of Zoology "P. Doderlein". Historical inventory number 148.
Rostrum on pedestal.
Length 44.4 cm.
• AN1330
Present in the historical collection, recovered in Palermo on an unknown date, stored at the Museum of Zoology "P. Doderlein". Historical inventory number 2590 bis.
Rostrum on pedestal. Currently in poor condition, with fat present all over the specimen.
Length 58.4 cm.

Genus *Pristis* Linck, 1790
Species *Pristis pectinata* Latham, 1794

• AN184
Present in the historical collection, recovered in Palermo between 1863-1880, stored at the Museum of Zoology "P. Doderlein". Historical inventory number 148.
Rostrum on pedestal.
Length 27.7 cm.
• AN185
Present in the historical collection, recovered in Palermo between 1863-1880, stored at the Museum of Zoology "P. Doderlein". Historical inventory number 148.
Rostrum on pedestal.
Length 116 cm.

Species *Pristis zijsron* Bleeker, 1851

• AN183
Present in the historical collection, recovered in Palermo between 1863-1880, stored at the Museum of Zoology "P. Doderlein". Historical inventory number 148.
Rostrum on pedestal.
Length 121.3 cm.
• AN186
Present in the historical collection, recovered in Palermo between 1863-1880, stored at the Museum of Zoology "P. Doderlein". Historical inventory number 148.
Rostrum on pedestal.
Length 132.7 cm.

Family Rhinobatidae Bonaparte, 1835
Genus *Rhinobatos* Linck, 1790
Species sp.

• AN652
Present in the historical collection, recovered in Palermo between 1863-1880, stored at the Museum of Zoology "P. Doderlein". Historical inventory number 151.
Digestive system on pedestal. Currently in poor condition.
Length 36.4 cm.

Species *Rhinobatos rhinobatos* Linnaeus, 1758

• AN14
Present in the historical collection, recovered in Palermo between 1863-1880, stored at the Museum of Zoology "P. Doderlein". Historical inventory number 139.
Skeleton on pedestal.
♂, juvenile, total length 60.8m.
• AN15
Present in the historical collection, recovered in Palermo on an unknown date, stored at the Museum of Zoology "P. Doderlein". Historical inventory number 2648 bis.
Skeleton on pedestal.
♀, juvenile, total length 77.4 cm.
• AN428
Present in the historical collection, recovered in Palermo between 1863-1880, stored at the Museum of Zoology "P. Doderlein". Historical inventory number 152.
Respiratory system on pedestal. Currently in poor condition.
Length 6.2 cm.
• AN544
Present in the historical collection, recovered in Palermo between 1863-1880, stored at the Museum of Zoology "P. Doderlein". Historical inventory number 151.
Digestive system on pedestal. Currently in poor condition, thoroughly damaged.
Length 29.1 cm.
• AN587
Present in the historical collection, recovered in Palermo between 1863-1880, stored at the Museum of Zoology "P. Doderlein". Historical inventory number 151.
Digestive system on pedestal. Currently in poor condition, thoroughly damaged.
Length 24.4 cm.
• P560
Present in the historical collection, bought in Palermo on the 31.12.1882 by Eredi di Paola, stored at the Museum of Zoology "P. Doderlein". Historical inventory number 565.
Taxidermied specimen on pedestal.
♀, juvenile, total length 45.9 cm.
• P589
Present in the historical collection, recovered in Palermo between 1863-1880, stored at the Museum of Zoology "P. Doderlein". Historical inventory number 121.
Taxidermied specimen on pedestal.
♂, juvenile, total length 69.1 cm.
• P590
Present in the historical collection, bought in Palermo on the 31.12.1882 by Eredi di Paola, stored at the Museum of Zoology "P. Doderlein". Historical inventory number 565.
Taxidermied specimen on pedestal.
♀, juvenile, total length 77.7 cm.
• P591
Present in the historical collection, bought in Palermo on the 30.04.1883 by Eredi di Paola, stored at the Museum of Zoology "P. Doderlein". Historical inventory number 600.

Taxidermied specimen on pedestal. Currently in poor condition, presence of white paint near the pelvic fins, not consistent with the taxidermy.
♀, sub-adult, Total length 93.1 cm.

Order Torpediniformes
Family Torpedinidae Henle, 1834
Genus *Tetronarce* Gill, 1862
Species *Tetronarce nobiliana* Bonaparte, 1835

• AN19
Present in the historical collection, recovered in Palermo between 1863-1880, stored at the Museum of Zoology "P. Doderlein". Historical inventory number 139.
Skeleton on pedestal.
♀, juvenile, total length 36.9 cm.
• P581
Present in the historical collection, recovered in Palermo between 1863-1880, stored at the Museum of Zoology "P. Doderlein". Historical inventory number 119.
Taxidermied specimen on pedestal.
♀, sub-adult, total length 107.7 cm.

Genus *Torpedo* Duméril, 1806
Species *Torpedo marmorata* Risso, 1810
• AN18
Present in the historical collection, recovered in Palermo between 1863-1880, stored at the Museum of Zoology "P. Doderlein". Historical inventory number 139.
Skeleton on pedestal. Currently in poor condition.
♀, sub-adult, total length 44.5 cm.
• PL46
Present in the historical collection, recovered in Palermo on an unknown date, stored at the Museum of Zoology "P. Doderlein". Historical inventory number unknown.
Specimen preserved in alcohol.
Total length 16 cm.
• P578
Present in the historical collection, bought in Palermo on the 16.08.1884 by Eredi Di Paola, stored at the Museum of Zoology "P. Doderlein". Historical inventory number 694.
Taxidermied specimen on pedestal.
♀, adult, total length 60.8 cm.
• P626
Present in the historical collection, recovered in Palermo between 1863-1880, stored at the Museum of Zoology "P. Doderlein". Historical inventory number 121.
Taxidermied specimen on pedestal.
♀, sub-adult, total length 40.7 cm.
• P629
Present in the historical collection, recovered in Palermo between 1863-1880, stored at the Museum of Zoology "P. Doderlein". Historical inventory number 119.
Taxidermied specimen on pedestal. Currently in poor condition.
♀, sub-adult, total length 48.5 cm.

Species *Torpedo torpedo* Linnaeus, 1758

• AN533

Present in the historical collection, recovered in Palermo between 1863-1880, stored at the Museum of Zoology "P. Doderlein". Historical inventory number 154.
Circulatory system on pedestal.
Length 9.5 cm.
• AN641
Present in the historical collection, recovered in Palermo between 1863-1880, stored at the Museum of Zoology "P. Doderlein". Historical inventory number 151.
Digestive system on pedestal.
Length 12.2 cm.
• PL74
Present in the historical collection, recovered in Palermo on an unknown date, stored at the Museum of Zoology "P. Doderlein". Historical inventory number unknown.
Specimen preserved in alcohol.
♀, juvenile, total length 11.3 cm.
• P575
Present in the historical collection, recovered in Palermo on an unknown date, stored at the Museum of Zoology "P. Doderlein". Historical inventory number 2645 bis.
Taxidermied specimen on pedestal.
♂, juvenile, total length 29.4 cm.
• P587
Present in the historical collection, recovered in Palermo between 1863-1880, stored at the Museum of Zoology "P. Doderlein". Historical inventory number 121.
Taxidermied specimen on pedestal.
♀, juvenile, total length 37.4 cm.
• P603
Present in the historical collection, recovered in Palermo on an unknown date, stored at the Museum of Zoology "P. Doderlein". Historical inventory number 2645 bis.
Taxidermied specimen on pedestal.
♀, juvenile, total length 34.3 cm

Superorder Selachimorpha
Order Hexanchiformes
Family Hexanchidae Gray, 1851
Genus *Hexanchus* Rafinesque, 1810
Species *Hexanchus griseus* Bonnaterre, 1788

• AN73
Present in the historical collection, recovered in Palermo between 1863-1880, stored at the Museum of Zoology "P. Doderlein". Historical inventory number 143.
Skull on pedestal.
Length 41.3 cm.
• AN89
Present in the historical collection, recovered in Palermo between 1863-1880, stored at the Museum of Zoology "P. Doderlein". Historical inventory number 147.
Jaw on pedestal.
Length 61.5 cm.
• AN98
Present in the historical collection, recovered in Palermo between 1863-1880, stored at the Museum of Zoology "P. Doderlein". Historical inventory number 147.
Jaw on pedestal.
Length 27.4 cm.
• AN106

Present in the historical collection, recovered in Palermo between 1863-1880, stored at the Museum of Zoology "P. Doderlein". Historical inventory number 147.
Jaw on pedestal.
Length 35.2 cm.
• AN566
Present in the historical collection, recovered in Palermo between 1863-1880, stored at the Museum of Zoology "P. Doderlein". Historical inventory number 151.
Digestive system on pedestal.
Length 49.3 cm.
• P524
Present in the historical collection, recovered in Palermo between 1863-1880, stored at the Museum of Zoology "P. Doderlein". Historical inventory number 119.
Taxidermied specimen on pedestal.
♀, juvenile, Total length 157.9 cm.

Genus *Heptranchias* Rafinesque, 1810
Species *Heptranchias perlo* Bonnaterre, 1788

• AN28
Present in the historical collection, recovered in Palermo between 1863-1880, stored at the Museum of Zoology "P. Doderlein". Historical inventory number 143.
Skull on pedestal.
Length 27.3 cm.
• AN49
Present in the historical collection, recovered in Palermo between 1863-1880, stored at the Museum of Zoology "P. Doderlein". Historical inventory number 139.
Skeleton on pedestal.
♂, sub-adult, total length 83.1 cm.
• P539
Present in the historical collection, recovered in Palermo between 1863-1880, stored at the Museum of Zoology "P. Doderlein". Historical inventory number 120.
Taxidermied specimen on pedestal.
♂, sub-adult, Total length 84.5 cm.
• P540
Present in the historical collection, recovered in Palermo between 1863-1880, stored at the Museum of Zoology "P. Doderlein". Historical inventory number 120.
Taxidermied specimen on pedestal.
♂, adult, Total length 89.9 cm.
• P541
Present in the historical collection, recovered in Palermo between 1863-1880, stored at the Museum of Zoology "P. Doderlein". Historical inventory number 120.
Taxidermied specimen on pedestal.
♂, adult, Total length 99.2 cm.

Order Echinorhiniformes
Family Echinorhinidae Gill, 1862
Genus *Echinorhinus* Blainville, 1816
Species *Echinorhinus brucus* Bonnaterre, 1788

• AN50
Present in the historical collection, recovered in Palermo between 1863-1880, stored at the Museum of Zoology "P. Doderlein". Historical inventory number 143.

Splanchnocranium on pedestal.
Length 25.5 cm.
• AN95
Present in the historical collection, recovered in Palermo between 1863-1880, stored at the Museum of Zoology "P. Doderlein". Historical inventory number 147.
Jaw on pedestal.
Length 29.9 cm.
• AN110
Present in the historical collection, recovered in Palermo between 1863-1880, stored at the Museum of Zoology "P. Doderlein". Historical inventory number 147.
Jaw on pedestal.
Length 25.4 cm.
• AN143
Present in the historical collection, bought in Palermo on the 30.04.1883 by, stored at the Museum of Zoology "P. Doderlein". Historical inventory number 595.
Jaw on pedestal.
Length 12.5 cm.
• P517
Present in the historical collection, recovered in Palermo between 1863-1880, stored at the Museum of Zoology "P. Doderlein". Historical inventory number 119.
Taxidermied specimen on pedestal.
♂, juvenile, Total length 126.5 cm.

Order squaliformes
Family Centrophoridae Bleeker, 1859
Genus *Centrophorus* Müller & Henle, 1837
Species *Centrophorus granulosus* Bloch & Schneider, 1801

• AN130
Present in the historical collection, recovered in Palermo between 1863-1880, stored at the Museum of Zoology "P. Doderlein". Historical inventory number 143.
Skull on pedestal.
Length 9,8 cm
• AN557
Present in the historical collection, recovered in Palermo between 1863-1880, stored at the Museum of Zoology "P. Doderlein". Historical inventory number 151.
Digestive system on pedestal.
Length 41,2 cm.

Species *Centrophorus uyato* Rafinesque, 1810

• AN42
Present in the historical collection, recovered in Palermo between 1863-1880, stored at the Museum of Zoology "P. Doderlein". Historical inventory number 139.
Skull on pedestal.
♀, adult, length 95.7 cm.
• AN119
Present in the historical collection, recovered in Palermo between 1863-1880, stored at the Museum of Zoology "P. Doderlein". Historical inventory number 143.
Splanchnocranium on pedestal.
Length 11.3 cm.
• AN122

Present in the historical collection, recovered in Palermo between 1863-1880, stored at the Museum of Zoology "P. Doderlein". Historical inventory number 147.
Jaw on pedestal.
Length 8.4 cm.
• AN129
Present in the historical collection, recovered in Palermo between 1863-1880, stored at the Museum of Zoology "P. Doderlein". Historical inventory number 143.
Skull specimen on pedestal.
Length 9.2 cm.
• P532
Present in the historical collection, recovered in Palermo between 1863-1880, stored at the Museum of Zoology "P. Doderlein". Historical inventory number 119.
Taxidermied specimen on pedestal.
♀, sub-adult, Total length 84.4 cm.
• P538
Present in the historical collection, recovered in Palermo between 1863-1880, stored at the Museum of Zoology "P. Doderlein". Historical inventory number 119.
Taxidermied specimen on pedestal.
♂, sub-adult, Total length 77.4 cm.
• P632
Present in the historical collection, recovered in Palermo between 1863-1880, stored at the Museum of Zoology "P. Doderlein". Historical inventory number 120.
Taxidermied specimen on pedestal.
♂, juvenile, Total length 53.3 cm.
• P634
Present in the historical collection, recovered in Palermo on an unknown date, stored at the Museum of Zoology "P. Doderlein". Historical inventory number 2544 bis.
Taxidermied specimen on pedestal.
♀, juvenile, Total length 53.2 cm.

Family Dalatiidae Gray, 1851
Genus *Dalatias* Rafinesque, 1810
Species *Dalatias licha* Bonnaterre, 1788

• AN44
Present in the historical collection, recovered in Palermo on an unknown date, stored at the Museum of Zoology "P. Doderlein". Historical inventory number 2648 bis.
Skeleton specimen on pedestal.
♂, total length 77.3 cm.
• AN61
Present in the historical collection, recovered in Palermo between 1863-1880, stored at the Museum of Zoology "P. Doderlein". Historical inventory number 143.
Splanchnocranium on pedestal.
♂, length 10.2 cm.
• AN125
Present in the historical collection, recovered in Palermo between 1863-1880, stored at the Museum of Zoology "P. Doderlein". Historical inventory number 143.
Jaw on pedestal.
Length 9.2 cm.
• AN550
Present in the historical collection, recovered in Palermo between 1863-1880, stored at the Museum of Zoology "P. Doderlein". Historical inventory number 151.

Digestive system on pedestal. Currently in mediocre condition, it lost colour in some points.
Length 36,9 cm.

- AN643

Present in the historical collection, recovered in Palermo between 1863-1880, stored at the Museum of Zoology "P. Doderlein". Historical inventory number 151.
Digestive system on pedestal.
Length 28,4 cm.

- AN659

Present in the historical collection, recovered in Palermo between 1863-1880, stored at the Museum of Zoology "P. Doderlein". Historical inventory number 151.
Digestive system on pedestal.
Length 32.3 cm.

- P519

Present in the historical collection, bought in Palermo on the 31.03.1885 by Eredi di Paola, stored at the Museum of Zoology "P. Doderlein". Historical inventory number 743.
Taxidermied specimen on pedestal
♀, juvenile, total length 47.3 cm.

- P521

Present in the historical collection, recovered in Palermo on an unknown date, stored at the Museum of Zoology "P. Doderlein". Historical inventory number 2542 bis.
Taxidermied specimen on pedestal
♀, sub-adult, total length 70.5 cm.

- P529

Present in the historical collection, recovered in Palermo between 1863-1880, stored at the Museum of Zoology "P. Doderlein". Historical inventory number 120.
Taxidermied specimen on pedestal
♀, sub-adult, total length 93.3 cm.

- P530

Present in the historical collection, recovered in Palermo between 1863-1880, stored at the Museum of Zoology "P. Doderlein". Historical inventory number 120.
Taxidermied specimen on pedestal.
♀, sub-adult, total length 98.5 cm.

Family Etmopteridae Fowler, 1934
Genus *Etmopterus* Rafinesque, 1810
Species *Etmopterus spinax* Linnaeus, 1758

- AN27

Present in the historical collection, recovered in Palermo between 1863-1880, stored at the Museum of Zoology "P. Doderlein". Historical inventory number 139.
Skeleton on pedestal.
♀, sub-adult, total length 37.6 cm.

- AN629

Present in the historical collection, recovered in Palermo between 1863-1880, stored at the Museum of Zoology "P. Doderlein". Historical inventory number 151.
Digestive system on pedestal. Currently in mediocre condition, presence of a small hole.
Length 9.2 cm.

- P525

Present in the historical collection, recovered in Palermo between 1863-1880, stored at the Museum of Zoology "P. Doderlein". Historical inventory number 121.
Taxidermied specimen on pedestal.
♂, adult, total length 38.7 cm.

• P526
Present in the historical collection, recovered in Palermo between 1863-1880, stored at the Museum of Zoology "P. Doderlein". Historical inventory number 121.
Taxidermied specimen on pedestal.
♂, adult, total length 42.2 cm.

Family Oxynotidae Gill, 1863
Genus *Oxynotus* Rafinesque, 1810
Species *Oxynotus centrina* Linnaeus, 1758

• AN46
Present in the historical collection, recovered in Palermo between 1863-1880, stored at the Museum of Zoology "P. Doderlein". Historical inventory number 139.
Skeleton on pedestal.
♀, sub-adult, total length 64.4 cm.
• P515
Present in the historical collection, recovered in Palermo between 1863-1880, stored at the Museum of Zoology "P. Doderlein". Historical inventory number 120.
Taxidermied specimen on pedestal.
♀, sub-adult, total length 64.3 cm.
• P516
Present in the historical collection, recovered in Palermo on an unknown date, stored at the Museum of Zoology "P. Doderlein". Historical inventory number 2543 bis.
Taxidermied specimen on pedestal.
♀, sub-adult, total length 65 cm.

Family Somniosidae Jordan, 1888
Genus *Somniosus* Lesueur, 1818
Species *Somniosus rostratus* Risso, 1827

• P528
Present in the historical collection, recovered in Palermo between 1863-1880, stored at the Museum of Zoology "P. Doderlein". Historical inventory number 120.
Taxidermied specimen on pedestal.
♀, sub-adult, total length 82.2 cm.

Family Squalidae de Blainville, 1816
Genus *Squalus* Linnaeus, 1758
Species *Squalus blainville* Risso, 1827

• AN41
Present in the historical collection, recovered in Palermo between 1863-1880, stored at the Museum of Zoology "P. Doderlein". Historical inventory number 139.
Taxidermied specimen on pedestal.
♂, adult, total length 95.3 cm.
• P631
Present in the historical collection, recovered in Palermo between 1863-1880, stored at the Museum of Zoology "P. Doderlein". Historical inventory number 120.
Taxidermied specimen on pedestal.
♀, adult, total length 91.3 cm.
• P633

Present in the historical collection, recovered in Palermo between 1863-1880, stored at the Museum of Zoology "P. Doderlein". Historical inventory number 120.
Taxidermied specimen on pedestal.
♂, adult, total length 66.4 cm.

Species *Squalus acanthias* Linnaeus, 1758
• AN561
Present in the historical collection, recovered in Palermo between 1863-1880, stored at the Museum of Zoology "P. Doderlein". Historical inventory number 151.
Digestive system on pedestal. Currently in poor condition, fractured into two sections.
Length 27.4 cm.

Order Squatiniformes
Family Squatinidae de Blainville, 1816
Genus *Squatina* Duméril, 1805
Species *Squatina aculeata* Cuvier, 1829

• AN83
Present in the historical collection, recovered in Palermo between with unknown date, stored at the Museum of Zoology "P. Doderlein". Historical inventory number 2650 bis.
Jaw on pedestal.
Length 21.7 cm
• AN140
Present in the historical collection, recovered in Palermo between 1863-1880, stored at the Museum of Zoology "P. Doderlein". Historical inventory number 147.
Jaw on pedestal.
Length 23.1 cm.
• AN142
Present in the historical collection, recovered in Palermo between 1863-1880, stored at the Museum of Zoology "P. Doderlein". Historical inventory number 143.
Jaw on pedestal.
Length 19.5 cm.
• AN405
Present in the historical collection, recovered in Palermo between with unknown date, stored at the Museum of Zoology "P. Doderlein". Historical inventory number 2050 bis.
Jaw on pedestal.
Length 17.9 cm.
• P561
Present in the historical collection, recovered in Palermo between 1863-1880, stored at the Museum of Zoology "P. Doderlein". Historical inventory number 120.
Taxidermied specimen on pedestal.
♀, adult, total length 81.2 cm.
• P563
Present in the historical collection, recovered in Palermo between 1863-1880, stored at the Museum of Zoology "P. Doderlein". Historical inventory number 121.
Taxidermied specimen on pedestal.
♂, juvenile, total length 27.4 cm.

Species *Squatina oculata* Bonaparte, 1840

• AN33

Present in the historical collection, recovered in Palermo between with unknown date, stored at the Museum of Zoology "P. Doderlein". Historical inventory number 2648 bis.
Skeleton on pedestal.
♀, sub-adult, total length 72.9 cm.
• AN84
Present in the historical collection, recovered in Palermo between with unknown date, stored at the Museum of Zoology "P. Doderlein". Historical inventory number 2650 bis.
Jaw on pedestal.
Length 19.9 cm.
• PL125
Present in the historical collection, recovered in Palermo between with unknown date, stored at the Museum of Zoology "P. Doderlein". Historical inventory number unknown.
Two specimens preserved in alcohol.
♀, juveniles, total length 32.3 cm.
• P564
Present in the historical collection, recovered in Palermo on the 30.04.1882, stored at the Museum of Zoology "P. Doderlein". Historical inventory number 488.
Taxidermied specimen on pedestal.
♀, adult, total length 101.2 cm.

Species *Squatina squatina* Linnaeus, 1758

• AN31
Present in the historical collection, recovered in Palermo between 1863-1880, stored at the Museum of Zoology "P. Doderlein". Historical inventory number 139.
Skeleton on pedestal.
♀, adult, total length 91.3 cm.
• AN32
Present in the historical collection, recovered in Palermo between with unknown date, stored at the Museum of Zoology "P. Doderlein". Historical inventory number 2650 bis.
Jaw on pedestal.
Length 13.8 cm.
• AN656
Present in the historical collection, recovered in Palermo between 1863-1880, stored at the Museum of Zoology "P. Doderlein". Historical inventory number 151.
Digestive system on pedestal.
Length 21.5 cm.
• P658
Present in the historical collection, recovered in Palermo on the 30.04.1882, stored at the Museum of Zoology "P. Doderlein". Historical inventory number 488.
Taxidermied specimen on pedestal.
♂, adult, total length 160.1 cm.

Order Heterodontiformes
Family Heterodontidae Gray, 1851
Genus *Heterodontus* Blainville, 1816
Species *Heterodontus zebra* Gray, 1831

• AN1331
Present in the historical collection, donated from Natural History Museum of Milan by Bellotti, stored at the Museum of Zoology "P. Doderlein". Historical inventory number unknown.
Lower jaw on pedestal.

Length 10.2 cm.

Order Carcharhiniformes
Family Carcharhinidae Jordan & Evermann, 1896
Genus *Carcharhinus* Blainville, 1816
Species *Carcharhinus brachyurus* Günther, 1870

• AN80
Present in the historical collection, recovered in Palermo between 1863-1880, stored at the Museum of Zoology "P. Doderlein". Historical inventory number 147.
Jaw on pedestal.
Length 20.5 cm.
• AN359
Present in the historical collection, recovered in Palermo between 1863-1880, stored at the Museum of Zoology "P. Doderlein". Historical inventory number 147.
Jaw on pedestal.
Length 27.7 cm.

Species *Carcharhinus plumbeus* Nardo, 1827

• AN105
Present in the historical collection, recovered in Palermo between 1863-1880, stored at the Museum of Zoology "P. Doderlein". Historical inventory number 147.
Jaw on pedestal.
Length 15.7 cm.
• AN109
Present in the historical collection, bought in Palermo on the 31.12.1888 by Antonino Puleo, stored at the Museum of Zoology "P. Doderlein". Historical inventory number 975.
Jaw on pedestal.
Length 22.4 cm.
• AN111
Present in the historical collection, recovered in Palermo between 1863-1880, stored at the Museum of Zoology "P. Doderlein". Historical inventory number 147.
Jaw on pedestal.
Length 13.1 cm.
• AN117
Present in the historical collection, recovered in Palermo between 1863-1880, stored at the Museum of Zoology "P. Doderlein". Historical inventory number 147.
Jaw on pedestal.
Length 19.1 cm.
• AN123
Present in the historical collection, recovered in Palermo between 1863-1880, stored at the Museum of Zoology "P. Doderlein". Historical inventory number 147.
Jaw on pedestal.
Length 45.9 cm.
• AN131
Present in the historical collection, recovered in Palermo between 1863-1880, stored at the Museum of Zoology "P. Doderlein". Historical inventory number 147.
Jaw on pedestal.
Length 20.3 cm.
• AN144

Present in the historical collection, recovered in Palermo between 1863-1880, stored at the Museum of Zoology "P. Doderlein". Historical inventory number 147.
Jaw on pedestal.
Length 10.6 cm.
• P551
Present in the historical collection, recovered in Palermo between 1863-1880, stored at the Museum of Zoology "P. Doderlein". Historical inventory number 120.
Taxidermied specimen on pedestal.
♀, juvenile, total length 86.5 cm.
• P554
Present in the historical collection, recovered in Palermo between 1863-1880, stored at the Museum of Zoology "P. Doderlein". Historical inventory number 119.
Taxidermied specimen on pedestal.
♂, sub-adult, total length 155 cm.

Genus *Prionace* Cantor, 1849
Species *Prionace glauca* Linnaeus, 1758

• AN54
Present in the historical collection, recovered in Palermo between 1863-1880, stored at the Museum of Zoology "P. Doderlein". Historical inventory number 147.
Jaw on pedestal.
Length 15.2 cm.
• AN93
Present in the historical collection, recovered in Palermo with unknown date, stored at the Museum of Zoology "P. Doderlein". Historical inventory number unknown.
Jaw on pedestal.
Length 14.1 cm.
• AN100
Present in the historical collection, recovered in Palermo between 1863-1880 stored at the Museum of Zoology "P. Doderlein". Historical inventory number 147.
Jaw on pedestal.
Length 31.4 cm.
• AN118
Present in the historical collection, recovered in Palermo between 1863-1880, stored at the Museum of Zoology "P. Doderlein". Historical inventory number 147.
Jaw on pedestal.
Length 25.6 cm.
• AN1235
Present in the historical collection, recovered in Palermo with unknown date, stored at the Museum of Zoology "P. Doderlein". Historical inventory number unknown.
Jaw on pedestal.
Length 20.4 cm.
• PL390
Present in the historical collection, recovered in Palermo between with unknown date, stored at the Museum of Zoology "P. Doderlein". Historical inventory number unknown.
Two specimens preserved in alcohol.
♀, juvenile, total length 32.3 cm.
• P535
Present in the historical collection, recovered in Palermo between 1863-1880, stored at the Museum of Zoology "P. Doderlein". Historical inventory number 119.
Taxidermied specimen on pedestal.

♀, sub-adult, total length 143.5 cm.
• P537
Present in the historical collection, recovered in Palermo between 1863-1880, stored at the Museum of Zoology "P. Doderlein". Historical inventory number 121.
Taxidermied specimen on pedestal.
♀, juvenile, total length 53.2 cm.

Family Pentanchidae Smith, 1912
Genus *Galeus* Rafinesque, 1810
Species *Galeus melastomus* Rafinesque, 1810

• AN661
Present in the historical collection, recovered in Palermo between 1863-1880, stored at the Museum of Zoology "P. Doderlein". Historical inventory number 158.
Digestive system on pedestal.
Length 9.5 cm.

Family Scyliorhinidae Gill, 1862
Genus *Scyliorhinus* Blainville, 1816
Species *Scyliorhinus canicula* Linnaeus, 1758

• AN75
Present in the historical collection, recovered in Palermo between 1863-1880, stored at the Museum of Zoology "P. Doderlein". Historical inventory number 143.
Skull on pedestal.
Length 9.8 cm.
• P536
Present in the historical collection, recovered in Palermo between 1863-1880, stored at the Museum of Zoology "P. Doderlein". Historical inventory number 121.
Taxidermied specimen on pedestal.
♀, adult, total length 42.9 cm.
• P542
Present in the historical collection, recovered in Palermo on the 08.05.1882, stored at the Museum of Zoology "P. Doderlein". Historical inventory number 502.
Taxidermied specimen on pedestal.
♀, adult, total length 64.4 cm.
• P543
Present in the historical collection, recovered in Palermo between 1863-1880, stored at the Museum of Zoology "P. Doderlein". Historical inventory number 120.
Taxidermied specimen on pedestal.
♀, adult, total length 70.2 cm.
• P544
Present in the historical collection, recovered in Palermo between 1863-1880, stored at the Museum of Zoology "P. Doderlein". Historical inventory number 120.
Taxidermied specimen on pedestal.
♀, adult, total length 73.3 cm.

Species *Scyliorhinus stellaris* Linnaeus, 1758

• AN43
Present in the historical collection, recovered in Palermo between 1863-1880, stored at the Museum of Zoology "P. Doderlein". Historical inventory number 139.

Skeleton on pedestal.
♂, sub-adult, total length 80.7 cm.
• AN135
Present in the historical collection, recovered in Palermo between 1863-1880, stored at the Museum of Zoology "P. Doderlein". Historical inventory number 143.
Skull on pedestal.
Length 11.9 cm.

Family Sphyrnidae Bonaparte, 1840
Genus *Sphyrna* Rafinesque, 1810
Species *Sphyrna zygaena* Linnaeus, 1758

• AN48
Present in the historical collection, recovered in Palermo between 1863-1880, stored at the Museum of Zoology "P. Doderlein". Historical inventory number 139.
Skeleton on pedestal.
♂, juvenile, total length 47.4 cm.
• AN91
Present in the historical collection, recovered in Palermo between 1863-1880, stored at the Museum of Zoology "P. Doderlein". Historical inventory number 143.
Skull on pedestal.
Length 33.9 cm.
• AN97
Present in the historical collection, recovered in Palermo between 1863-1880, stored at the Museum of Zoology "P. Doderlein". Historical inventory number 143.
Skull on pedestal.
Length 49.4 cm.
• AN133
Present in the historical collection, recovered in Palermo between 1863-1880, stored at the Museum of Zoology "P. Doderlein". Historical inventory number 143.
Skull on pedestal.
Length 30.5 cm.
• AN547
Present in the historical collection, recovered in Palermo between 1863-1880, stored at the Museum of Zoology "P. Doderlein". Historical inventory number 151.
Digestive system on pedestal.
Length 36.5 cm.
• AN579
Present in the historical collection, recovered in Palermo between 1863-1880, stored at the Museum of Zoology "P. Doderlein". Historical inventory number 151.
Digestive system on pedestal.
Length 10.7 cm.
• PL 283
Present in the historical collection, recovered in Palermo with unknown date, stored at the Museum of Zoology "P. Doderlein". Historical inventory number unknown.
Two specimens preserved in alcohol.
♂, ♀, juveniles, total length 43.1 cm.
• P556
Present in the historical collection, recovered in Palermo between 1863-1880, stored at the Museum of Zoology "P. Doderlein". Historical inventory number 121.
Taxidermied specimen on pedestal.
♂, juvenile, total length 56.5 cm.

• P557
Present in the historical collection, recovered in Palermo between 1863-1880, stored at the Museum of Zoology "P. Doderlein". Historical inventory number 121.
Taxidermied specimen on pedestal.
♀, juvenile, total length 58 cm.
• P558
Present in the historical collection, recovered in Palermo between 1863-1880, stored at the Museum of Zoology "P. Doderlein". Historical inventory number 120.
Taxidermied specimen on pedestal.
♂, sub-adult, total length 83.5 cm.
• P559
Present in the historical collection, recovered in Palermo between 1863-1880, stored at the Museum of Zoology "P. Doderlein". Historical inventory number 119.
Taxidermied specimen on pedestal.
♂, adult, total length 178.5 cm.

Family Triakidae Gray, 1851
Genus *Galeorhinus* de Blainville, 1816
Species *Galeorhinus galeus* Linnaeus, 1758

• AN124
Present in the historical collection, recovered in Palermo on the 31.07.1885, stored at the Museum of Zoology "P. Doderlein". Historical inventory number 763.
Jaw on pedestal.
Length 15 cm.
• AN134
Present in the historical collection, recovered in Palermo between 1863-1880, stored at the Museum of Zoology "P. Doderlein". Historical inventory number 143.
Skull on pedestal.
Length 16.9 cm.
• AN139
Present in the historical collection, recovered in Palermo between 1863-1880, stored at the Museum of Zoology "P. Doderlein". Historical inventory number 143.
Skull on pedestal.
Length 12.7 cm.
• P527
Present in the historical collection, recovered in Palermo between 1863-1880, stored at the Museum of Zoology "P. Doderlein". Historical inventory number 120.
Taxidermied specimen on pedestal.
♂, total length 130.2 cm.
• P531
Present in the historical collection, recovered in Palermo between 1863-1880, stored at the Museum of Zoology "P. Doderlein". Historical inventory number 120.
Taxidermied specimen on pedestal.
♀, total length 80.7 cm.

Genus *Mustelus* Linck, 1790
Species *Mustelus asterias* Cloquet, 1821

• AN40
Present in the historical collection, recovered in Palermo with unknown date, stored at the Museum of Zoology "P. Doderlein". Historical inventory number 2648 bis.

Skeleton on pedestal.
♂, sub-adult, total length 109.7 cm
• AN1238
Present in the historical collection, recovered in between 1863-1880, stored at the Museum of Zoology "P. Doderlein". Historical inventory number 143.
Jaw on pedestal.
Length 11.4 cm.
• PL?
Present in the historical collection, recovered in Palermo with unknown date, stored at the Museum of Zoology "P. Doderlein". Historical inventory number unknown.
Specimen preserved in alcohol.
Total length 39.3 cm.
• P534
Present in the historical collection, recovered in Palermo with unknown date, stored at the Museum of Zoology "P. Doderlein". Historical inventory number unknown.
Taxidermied specimen on pedestal.
♀, adult, total length 125.6 cm.

Species *Mustelus mustelus* Linnaeus, 1758

• AN59
Present in the historical collection, recovered in Palermo between 1863-1880, stored at the Museum of Zoology "P. Doderlein". Historical inventory number 143.
Splanchnocranium on pedestal.
Length 26.5 cm.
• AN636
Present in the historical collection, recovered in Palermo between 1863-1880, stored at the Museum of Zoology "P. Doderlein". Historical inventory number 151.
Digestive system on pedestal.
Length 37.4 cm.
• P520
Present in the historical collection, recovered in Palermo between 1863-1880, stored at the Museum of Zoology "P. Doderlein". Historical inventory number 121.
Taxidermied specimen on pedestal.
♀, juvenile, total length 69.5 cm.
• P533
Present in the historical collection, recovered in Palermo between 1863-1880 stored at the Museum of Zoology "P. Doderlein". Historical inventory number 120.
Taxidermied specimen on pedestal.
♀, sub-adult, total length 95.2 cm

Order Lamniformes
Family Alopiidae Bonaparte, 1835
Genus *Alopias* Rafinesque, 1810
Species *Alopias vulpinus* Bonnaterre, 1788

• AN52
Present in the historical collection, recovered in Palermo between 1863-1880, stored at the Museum of Zoology "P. Doderlein". Historical inventory number 147.
Jaw on pedestal.
Length 15,4 cm.

• AN53
Present in the historical collection, recovered in Palermo between 1863-1880, stored at the Museum of Zoology "P. Doderlein". Historical inventory number 147.
Jaw on pedestal.
Length 20,3 cm.
• AN72
Present in the historical collection, recovered in Palermo on the 31.12.1888, stored at the Museum of Zoology "P. Doderlein". Historical inventory number 974.
Splanchnocranium on pedestal.
Length 32,3 cm.
• AN79
Present in the historical collection, recovered in Palermo between 1863-1880, stored at the Museum of Zoology "P. Doderlein". Historical inventory number 143.
Skull on pedestal.
Length 9,5 cm.
• AN508
Present in the historical collection, bought in Palermo on the 21.12.1890 by Teodosio Destefani, stored at the Museum of Zoology "P. Doderlein". Historical inventory number 1120.
Ocular apparatus on pedestal.
Length 22.4 cm
• P552
Present in the historical collection, recovered in Palermo between 1863-1880, stored at the Museum of Zoology "P. Doderlein". Historical inventory number 119.
Taxidermied specimen on pedestal.
♂, juvenile, total length 132,4 cm.
• P553
Present in the historical collection, recovered in Palermo between 1863-1880, stored at the Museum of Zoology "P. Doderlein". Historical inventory number 119.
Taxidermied specimen on pedestal.
♂, juvenile, total length 174,2 cm.

Family Cetorhinidae Gill, 1861
Genus *Cetorhinus* Blainville, 1816
Species *Cetorhinus maximus* Gunnerus, 1765

• AN1410
Present in the historical collection, recovered in Palermo on the 17.12.1928, stored at the Museum of Zoology "P. Doderlein". Historical inventory number 2453.
Skeleton.
Total length 256.2 cm.
• P657
Present in the historical collection, recovered in Palermo on an unknown date, stored at the Museum of Zoology "P. Doderlein". Historical inventory number unknown.
Taxidermied specimen on pedestal.
♀, sub-adult, total length 350 cm.

Family Lamnidae Bonaparte, 1835
Genus *Carcharodon* Smith, 1838
Species *Carcharodon carcharias* Linnaeus, 1758

• AN108

Present in the historical collection, recovered in Palermo between 1863-1880, stored at the Museum of Zoology "P. Doderlein". Historical inventory number 147.
Jaw on pedestal.
Length 25.5 cm.

- AN115

Present in the historical collection, recovered in Palermo between 1863-1880, stored at the Museum of Zoology "P. Doderlein". Historical inventory number 147.
Jaw on pedestal.
Length 37.6 cm.

- AN128

Present in the historical collection, recovered in Palermo between 1863-1880, stored at the Museum of Zoology "P. Doderlein". Historical inventory number 147.
Jaw on pedestal.
Length 65.5 cm.

- AN145

Present in the historical collection, bought in Palermo on the 01.03.1893 by Prof. Pietro Doderlein, stored at the Museum of Zoology "P. Doderlein". Historical inventory number 1270.
Jaw on pedestal.
Length 50.6 cm.

Genus *Isurus* Rafinesque, 1810
Species *Isurus oxyrinchus* Rafinesque, 1810

- AN55

Present in the historical collection, recovered in Palermo between 1863-1880, stored at the Museum of Zoology "P. Doderlein". Historical inventory number 147.
Jaw on pedestal.
Length 23.3 cm.

- AN63

Present in the historical collection, recovered in Palermo between 1863-1880, stored at the Museum of Zoology "P. Doderlein". Historical inventory number 143.
Skull on pedestal.
Length 24.4 cm.

- AN64

Present in the historical collection, recovered in Palermo between 1863-1880, stored at the Museum of Zoology "P. Doderlein". Historical inventory number 147.
Jaw on pedestal.
Length 28.1 cm.

- AN65

Present in the historical collection, recovered in Palermo between 1863-1880, stored at the Museum of Zoology "P. Doderlein". Historical inventory number 147.
Jaw on pedestal.
Length 21.5 cm.

- AN77

Present in the historical collection, recovered in Palermo between 1863-1880, stored at the Museum of Zoology "P. Doderlein". Historical inventory number 147.
Jaw on pedestal.
Length 18.5 cm.

- AN90

Present in the historical collection, recovered in Palermo between 1863-1880, stored at the Museum of Zoology "P. Doderlein". Historical inventory number 147.
Jaw on pedestal.

Length 31.8 cm.
• AN101
Present in the historical collection, recovered in Palermo between 1863-1880, stored at the Museum of Zoology "P. Doderlein". Historical inventory number 147.
Jaw on pedestal.
Length 24.5 cm.
• AN141
Present in the historical collection, recovered in Palermo between 1863-1880, stored at the Museum of Zoology "P. Doderlein". Historical inventory number 143.
Splanchnocranium on pedestal.
Length 18.8 cm.
• AN1234
Present in the historical collection, recovered in Palermo on an unknown date, stored at the Museum of Zoology "P. Doderlein". Historical inventory number unknown.
Jaw on pedestal.
Length 22.8 cm.
• P547
Present in the historical collection, recovered in Palermo between on the 08.05.1882, stored at the Museum of Zoology "P. Doderlein". Historical inventory number 500.
Taxidermied specimen on pedestal.
♀, juvenile, total length 87.3 cm.
• P548
Present in the historical collection, recovered in Palermo between 1863-1880, stored at the Museum of Zoology "P. Doderlein". Historical inventory number 119.
Taxidermied specimen on pedestal.
♀, juvenile, total length 120.4 cm.

Genus *Lamna* Cuvier, 1816
Species *Lamna nasus* Bonnaterre, 1788

• AN112
Present in the historical collection, recovered in Palermo on the 05.08.1882, stored at the Museum of Zoology "P. Doderlein". Historical inventory number 501.
Jaw on pedestal.
Length 15.3 cm.

Family Odontaspididae Müller & Henle, 1839
Genus *Carcharias* Rafinesque, 1810
Species *Carcharias taurus* Rafinesque, 1810

• AN38
Present in the historical collection, recovered in Palermo between 1863-1880, stored at the Museum of Zoology "P. Doderlein". Historical inventory number 139.
Skeleton on pedestal.
♂, juvenile, total length 141.5 cm.
• AN60
Present in the historical collection, recovered in Palermo on the 30.04.1883, stored at the Museum of Zoology "P. Doderlein". Historical inventory number 602.
Skull on pedestal.
Length 11.6 cm.
• AN68

Present in the historical collection, recovered in Palermo between 1863-1880, stored at the Museum of Zoology "P. Doderlein". Historical inventory number 147.
Jaw on pedestal.
Length 19.4 cm.
• AN132
Present in the historical collection, recovered in Palermo between 1863-1880, stored at the Museum of Zoology "P. Doderlein". Historical inventory number 147.
Jaw on pedestal.
Length 11.5 cm.
• AN548
Present in the historical collection, recovered in Palermo between 1863-1880, stored at the Museum of Zoology "P. Doderlein". Historical inventory number 151.
Digestive system on pedestal.
Length 33.4 cm.
• P518
Present in the historical collection, recovered in Palermo between 1863-1880 stored at the Museum of Zoology "P. Doderlein". Historical inventory number 119.
Taxidermied specimen on pedestal.
♂, juvenile, total length 166.4 cm.
• P522
Present in the historical collection, recovered in Palermo between 1863-1880, stored at the Museum of Zoology "P. Doderlein". Historical inventory number 120.
Taxidermied specimen on pedestal.
♀, juvenile, total length 99.4 cm.

Genus *Odontaspis* Agassiz, 1838
Species *Odontaspis ferox* Risso, 1810

• AN57
Present in the historical collection, recovered in Palermo between 1863-1880 stored at the Museum of Zoology "P. Doderlein". Historical inventory number 143.
Splanchnocranium on pedestal.
Length 31.5 cm.
• AN66
Present in the historical collection, recovered in Palermo between 1863-1880, stored at the Museum of Zoology "P. Doderlein". Historical inventory number 147.
Jaw on pedestal.
Length 26.4 cm.
• AN70
Present in the historical collection, recovered in Palermo between 1863-1880, stored at the Museum of Zoology "P. Doderlein". Historical inventory number 147.
Jaw on pedestal.
Length 34.1 cm.
• AN85
Present in the historical collection, recovered in Palermo between 1863-1880, stored at the Museum of Zoology "P. Doderlein". Historical inventory number 147.
Jaw on pedestal.
Length 18.8 cm.
• AN86
Present in the historical collection, recovered in Palermo on the 11.06.1886, stored at the Museum of Zoology "P. Doderlein". Historical inventory number 811.
Jaw on pedestal.

Length 17.9 cm.
• AN94
Present in the historical collection, recovered in Palermo between 1863-1880, stored at the Museum of Zoology "P. Doderlein". Historical inventory number 147.
Jaw on pedestal.
Length 22.7 cm.
• AN113
Present in the historical collection, recovered in Palermo between 1863-1880 stored at the Museum of Zoology "P. Doderlein". Historical inventory number 147.
Jaw on pedestal.
Length 11.6 cm.
• AN578
Present in the historical collection, recovered in Palermo between 1863-1880, stored at the Museum of Zoology "P. Doderlein". Historical inventory number 151.
Digestive system on pedestal.
Length 37.4 cm.
• AN1236
Present in the historical collection, recovered in Palermo on an unknown date, stored at the Museum of Zoology "P. Doderlein". Historical inventory number unknown.
Jaw on pedestal.
Length 107 cm.
• P523
Present in the historical collection, recovered in Palermo between 1863-1880, stored at the Museum of Zoology "P. Doderlein". Historical inventory number 119.
Taxidermied specimen on pedestal.
♂, juvenile, total length 120.2 cm.
• P549
Present in the historical collection, recovered in Palermo between 1863-1880, stored at the Museum of Zoology "P. Doderlein". Historical inventory number 119.
Taxidermied specimen on pedestal.
♂, sub-adult, total length 175.1 cm.
