# Supplementary material for: Integrated Taxonomy and Species Diversity of the Historical Chondrichthyan Collection of the Zoology Museum “Pietro Doderlein” at the University of Palermo (Italy)
Source: Biology (Basel). 2025 Aug 26;14(9):1129. doi: 10.3390/biology14091129 (PMC12467001; doi:10.3390/biology14091129)
Supplement: Supplementary file 1 [file biology-14-01129-s001.zip › Supplementary material.pdf]

Supplementary text

Text S1. Short biography of Pietro Doderlein: academic career, scientific contributions, and zoological research.

Pietro Doderlein was born in Dubrovnik on the 2nd of February 1809. He graduated in Medicine and Surgery at the University of Padua. In 1836, driven by his passion for the natural sciences, he became a teaching assistant in Natural History at the University of Padua under Professor Tommaso Antonio Catullo, who referred to him as a friend and a tireless naturalist [9]. Successively, at the age of 30, he became Full Professor of Natural History at the University of Modena, where he developed his studies in geology and palaeontology [10]. During this period, he also demonstrated skills in identifying and cataloguing biological specimens, curating entire collections of Brazilian birds and modest collections of reptiles and molluscs, thanks to several significant donations from Luigi Bompani and Giuseppe Casari [11-12]. In 1862, Carlo Matteucci, the Minister of Education of the newly formed Kingdom of Italy, proposed to Pietro Doderlein the position of Professor of Zoology and Comparative Anatomy at the University of Palermo, with the condition of establish a museum as beautiful as the one he had revitalized in Modena. Doderlein was expected to bring with him the "Italian" spirit to Sicily, a land that, at the time, needed to forget centuries of Spanish domination and cultural/political influences. Thus, in 1863 the "Museo di Zoologia della Regia Università di Palermo" was officially established, derived from the "Gabinetto di Zoologia", founded the previous year [13-14].

Noteworthy examples of his important contribution in zoological pubblications include "Avifauna del Modenese e della Sicilia" [16] which is a commented catalogue of the various sedentary and migratory bird species found in the two regions [16] with further investigation on the ornithological and ichthyological aspects in both Ustica and Pantelleria islands [17]. He is the author of two significant syntheses on the vertebrate fauna of Sicily [18-19], furthermore he assessed the first record in the Mediterranean of the extremely rare *Acanthocybium solandri* (Cuvier, 1832) [20], as well as the description of distinctive zoological and anatomical features of one of the rarest fish species in the Mediterranean, *Lophotes cepedianus* (Giorna, 1803) [21]. Particularly noteworthy is Doderlein's collection of Mediterranean groupers, which he studied extensively, and which are still preserved in the museum. Among these, a prominent example is the Sicilian Grouper, *Epinephelus sicanus* [22-23]. Another significant contribution concerns the first description of *Pteridium armatum* [24] (a synonym of *Benthocometes robustus*) published a few months after the work of Goode and Bean (1886). Finally, in the later years of his career, he wrote about the discovery of *Callionymus phaeton* (Günther, 1861) in the waters of the Gulf of Palermo [25].

More details about the life and the scientific work of Pietro Doderlein can be found in "In ricordo di Pietro Doderlein (2 FEBBRAIO 1809 - 28 MARZO 1895)" [14].

Text S2. Description of specimen labelling systems resulting from successive re-cataloguing at the Zoology Museum of the University of Palermo.

Over the years, the collection housed at the Museum of Zoology "Pietro Doderlein" has undergone several re-cataloguing efforts, resulting in the

accumulation of multiple labels associated with individual specimens. Single or multiple labels may appear on a single specimen. The modern labels, created in the 1980s, are always present and include the collection category P or AN (indicating taxidermy or anatomical specimens, respectively), the current inventory number, the species' common and scientific name, and the specimen's capture locality (Figure S1a). In addition to the most recent label, two or three historical labels are often present on the specimens. Category 1 labels (45 mm x 25 mm) are handwritten, either in block letters (Figure S1b) or cursive (Figure S1c), dating back to the time when the specimen was first added to the collection. They typically report the scientific name, common Italian name, local (dialect) name, and the place of capture. When present, these labels are glued to the front of the wooden base of the specimens. When absent, they may have been lost over time or detached and preserved in dedicated holders to maintain their integrity. Category 2 labels (Figure S1d) are glued to the wooden base and completely covered with a layer of varnish, likely applied around 1960. Some of these bear the heading "Gabinetto di zoologia" (Zoology Cabinet), while others are labelled "Istituto di Zoologia e Anatomia Comparata" (Institute of Zoology and Comparative Anatomy), dating back to around 1862. In both cases, the historical inventory number is handwritten. Category 3 labels (Figure S1e) with variable dimensions, are usually attached to the animal with a string. Although rare, when present, they contain the specimen's scientific name, sometimes also including the place of origin and/or the year it was added to the collection.

Supplementary Figures

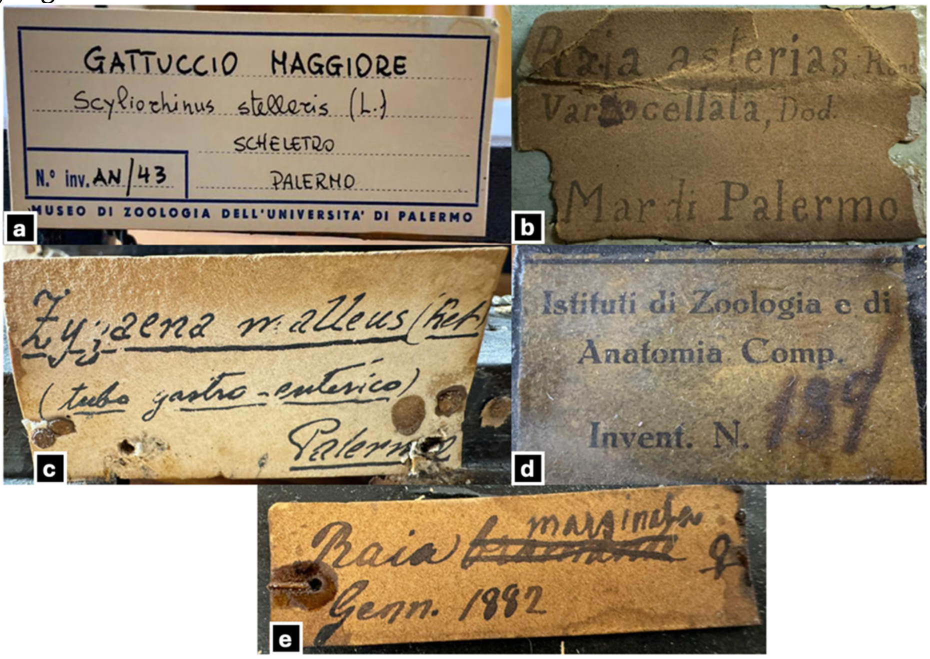

Figure S1. Example of different labels. (**a**) Modern label; (**b**) Historical label category 1 (block letters) (**c**) Historical label category 1 (cursive letters); (**d**) Historical label category 2; (**e**) Historical label category 3.

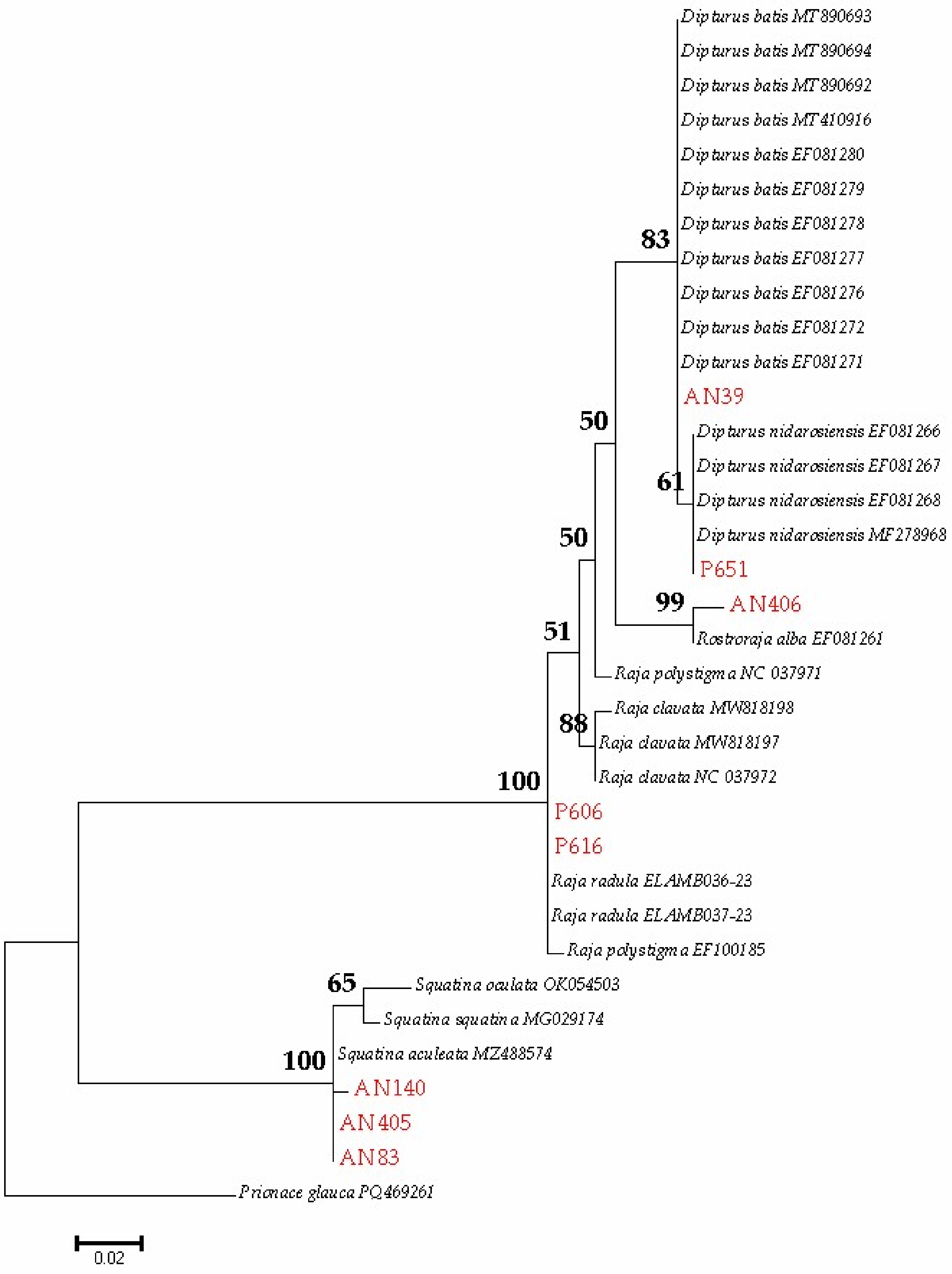
Dipturus batis MT890693
Dipturus batis MT890694
Dipturus batis MT890692
Dipturus batis MT410916
Dipturus batis EF081280
Dipturus batis EF081279
Dipturus batis EF081278
Dipturus batis EF081277
Dipturus batis EF081276
Dipturus batis EF081272
Dipturus batis EF081271
AN39
Dipturus nidarosiensis EF081266
Dipturus nidarosiensis EF081267
Dipturus nidarosiensis EF081268
Dipturus nidarosiensis MF278968
P651
AN406
Rostroraja alba EF081261
Raja polystigma NC 037971
Raja clavata MW818198
Raja clavata MW818197
Raja clavata NC 037972
P606
P616
Raja radula ELAMB036-23
Raja radula ELAMB037-23
Raja polystigma EF100185
Squatina oculata OK054503
Squatina squatina MG029174
Squatina aculeata MZ488574
AN140
AN405
AN83
Prionace glauca PQ469261
83
50
50
61
99
51
88
100
65
100
0.02

Figure S2. Molecular phylogenetic tree based on 216 bp of mitochondrial 12S sequences, reconstructed using the Maximum Likelihood method with the Tamura 3-parameter model. The tree with the highest log likelihood (711.96) is shown. Bootstrap support values (>50%) are indicated above the branches. The analysis included 8 sequences from this study (highlighted in red), 26 reference sequences from GenBank/BOLD, and 1 outgroup (*Prionace glauca*). The scale bar is given.

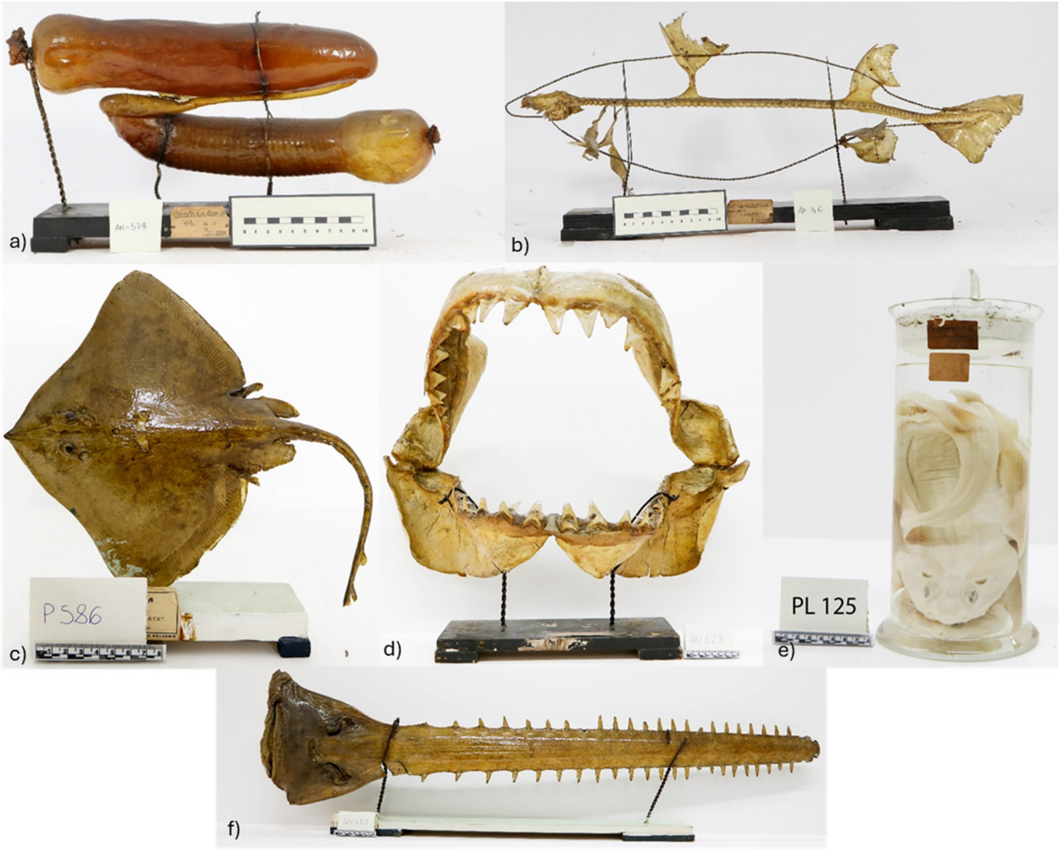

Figure S3. Examples of specimen conservation methods. (**a**) Stomachs and intestines prepared as dry specimens of *Odontaspis ferox;* (**b**) skeleton of *Oxynotus centrina*; (**c**) taxidermized specimens of *Raja polystigma*; (**d**) jaw of *Carcharodon carcharias*; (**e**) alcohol-formalin fixed liquid conservation of *Squatina oculata*; (**f**) rostrum of *Pristis pectinata.*

Supplementary Tables

Table S1. Historical code, record type and description of the historical code, extrapolated by the historical register.

CODE	RECORD	DESCRIPTION
118	Historical Record 1863–1880	Mari della Sicilia: Olocefali montati in pelle (mezzani)
119	Historical Record 1863–1880	Mari della Sicilia: Plagiostomi montati in pelle (grandi)
120	Historical Record 1863–1880	Mari della Sicilia: Plagiostomi montati in pelle (mezzani)
121	Historical Record 1863–1880	Mari della Sicilia: Plagiostomi montati in pelle (minori)
123	Historical Record 1863–1880	Mari della Sicilia: Testa isolata di Cephaloptera giorna
129	Historical Record 1863–1880	Plagiostomi
138	Historical Record 1863–1880	Scheletri di Olocefali
139	Historical Record 1863–1880	Scheletri di Plagiostomi
143	Historical Record 1863–1880	Teste intere di Pesci cartilaginei (crani)
147	Historical Record 1863–1880	Mascelle di Pesci cartilaginei
148	Historical Record 1863–1880	Rostri di Pesci Sega
151	Historical Record 1863–1880	Stomaci ed intestini copreparati a secco
152	Historical Record 1863–1880	Branchie copreparate a secco
153	Historical Record 1863–1880	Branchi Iniettate
154	Historical Record 1863–1880	Cuori copreparati a secco
158	Historical Record 1863–1880	Apparati di riproduzione preparati a secco
119	1882-04-08	Un grande pesce Plagiostomo montato in Pelle (*Odontaspis taurus*)
435	1881-07-31	Un Gattuccio (*Scyliorhinus canicula*)
439	1881-07-31	Diavolo cornuto, ovvero Cephal. Giona (testa)
478	1882-04-03	*Rhinobatus camiculus*
486	1882-04-30	Picara (mascella)
487	1882-04-30	Marzapane (testa)
488	1882-04-30	Due Pesce Squadro
489	1882-04-30	Raia (mascelle e visceri)
494	1882-04-30	Picara (mascella)
500	1882-05-08	*Lamna cornubica*
501	1882-05-08	*Lamna cornubica* (mascella*)*
502	1882-05-08	*Scyllium stellare (*giov. *acanthomotum)*
551	1882-12-19	Gigantesco vuglio/vugghio, ovvero *Trygon thalapsia* (testa e coda)
564	1882-12-31	Tre Picari (*Raia*)
565	1882-12-31	Due Pesce violino (*Rhinobatus columnae*)
567	1882-12-31	Picara (*Raia*)
589	1883-04-17	Una grossa razza (*Raja oxyrhynchus*)
594	1883-04-30	Pesce sorice (*Chimaera monstrosa*)
595	1883-04-30	Ficodindia, ovvero *Echin. spinosus* (mascella)
600	1883-04-30	Citarra (*Rhinobatus columnae*)
601	1883-04-30	Citarra (*Rhinobatus columnae*)
602	1883-04-30	Pesce cane, ovvero *Odontaspis taurus* (head)
638	1883-12-31	Ariolo, ovvero *Carcharodon lamia* (jaw)
658	1884-04-05	Picara pittusa (*Raja clavata*)
693	1884-08-16	Pesce violino, ovvero *Rhinobatos columnae* (head)
694	1884-08-16	Tremola (*Torpedo marmorata*)
695	1884-08-16	Picara (*Raja miraletus*)
709	1884-12-02	*Trygon thalassia* (grande)
710	1884-12-02	Due *Trygon* sp. (apparati femminili)

715	1884-12-16	Picara mantellina (*Pteroplatea altavela*)
716	1884-12-16	Picara liscia (*Raia chagrinea*)
741	1885-03-31	Razza (*Raja asterias* var.)
742	1885-03-31	Razza liscia (*R. bramante*)
743	1885-03-31	Pesce diavolo (*Scimnus lichia*)
744	1885-03-31	Razza quattrocchi (*R. radula*)
762	1885-07-31	Una Picaria liscia (*R. falsavela*)
763	1885-07-31	Pesce Cane (mascella)
765	1885-07-31	Una grande Tremula (*Torpedo nobiliana*)
803	1886-06-11	Due *Chimera monstrosa*
804	1886-06-11	Una *Rhinobatus columnae*
805	1886-06-11	Una *Raja flossada*
807	1886-06-11	Uno scheletro di *Pteroplatea altavela*
811	1886-06-11	*Odontapsis ferox* (mascella)
847	1886-12-31	Razza
852	1887-04-22	Pesce aquila, ovvero *Myliobatis bovina* (testa)
974	1888-12-31	Pesce sorce, ovvero *Alopias vulpes* (testa)
975	1888-12-31	Cagnesca, ovvero *Carcharius cania* (mascella)
1062	1890-02-14	Pesce ficundia (*Echinorhynus spinosus)*
1115	1890-12-21	*Rhinobatus columnae* (alcool)
1120	1890-12-21	*Alopias vulpes*
1270	1893-03-01	Grande pesce cane, ovvero *Carcharodon rondeletii* (mascella)
440-441	1881-07-31	Due piccole Crozze (*Zigaena malleus*)
737bis	1885-03-31	Razza
2542 bis	Additions up to 1922	*Scymnus licha* in pelle
2543 bis	Additions up to 1922	*Centrina salviani* in pelle
2544 bis	Additions up to 1922	*Acanthias ujatus* in pelle
2545 bis	Additions up to 1922	*Torpedo narce*
2590 bis	Additions up to 1922	*Raja asterias,* in alcool
2648 bis	Additions up to 1922	Scheletri incompleti di selaci
2650 bis	Additions up to 1922	Mascella di piccoli selaci

Table S2. Categories and subfields from the National catalogue of naturalistic heritage as indicated by Central Institute for the Cataloging and Documentation of Naturalistic Assets – Zoology [30] of the Ministry for Cultural Heritage and Activities. * Mandatory fields.

**CD CODE**
TSK Card type (BZN)*
LIR Research level (C)*
NCT UNIQUE CODE
NCTR Region code (R19)*
ESC Cataloging entity (UNIPA)*
ECP Competent authority (S88)*
**OG OBJECT**
OGT OBJECT*
OGTD Definition*
OGTV Identification*
OGTO Container type
OGTC Container name
QNT QUANTITY
QNTN Number
QNTI Quantity set
**SZ SYSTEMATICS - ZOOLOGY**
SZS SYSTEMATICS
SZSP Phylum*
SZSB Class
SZSO Order
SZSF Family
SZSR Genus
SZSS Species
SZSD Author and year
SZSG Degree of indetermination
SZSN Synonymous
SZA OTHER DATA
SZAS Sex
SZAE Age
SZE LABELS
SZEI Original heading
SZET Text
SZES Original data support
**LR COLLECTION DATA**
LRV COLLECTION LOCALITY
LRVT Type of localization*
LRVK Continent
LRVS State
LRVR Region
LRVP Province
LRVC Municipality
LRM Marine zoogeographical region
MAO Sea-Ocean

**LC GEOGRAPHICAL - ADMINISTRATIVE LOCATION**
PVC ACTUAL GEOGRAPHICAL - ADMINISTRATIVE LOCATION*
PVCS State*
PVCR Region*
PVCP Province*
PVCC Municipality*
LDC SPECIFIC COLLOCATION
LDCT Tipe
LDCQ Qualification
LDCN Denomination
LDCC Monumental complex of belonging
LDCU Denomination of the traffic space
LDCM Denomination of the conservation structure
**UB HERITAGE DATA AND COLLECTIONS**
INV INVENTORY
INVA Denomination
INVD Date*
INVC Collocation
INVN Number*
INVP Reference to the part
COL COLLECTION
COLD Denomination
COLC Collector name
COLI Item inventory number in the collection
**MT TECHNICAL DATA**
MTC TECHNIC AND MATERIAL
MIS MEASUREMENTS*
MISU Unit*
MISA Height
MISL Width
MISN Length
**DA ANALYTICAL DATA**
DES DESCRIPTION*
DESO Object*
NSC Historical and critical information
**CO CONSERVATION**
STC CONSERVATION STATE*
STCP Reference to the part
STCT Conservation type
STCE Specific of the conservation type
STCC Conservation state (Good/mediocre/bad)*
STCS Specific indication
**TU LEGAL CONDITION AND RESTRICTIONS**
ACQ ACQUISITION
ACQT Acquisition type*
ACQN Name
ACQD Acquisition date*
ACQL Acquisition place

CDG LEGAL CONDITION<br>CDGG Generical indication*<br>CDGS Specific indication<br>CDGI Address
**DO SOURCES AND REFERENCE DOCUMENTS**
FTAPHOTOGRAFIC DOCUMENTATION<br>FTAX Genus*<br>FTAP Type*<br>FTAA Author<br>FTAD Date<br>FNT SOURCES AND DOCUMENTS<br>FNTX Genus*<br>FNTP Type*<br>FNTA Authore<br>FNTT Denomination<br>FNTD Date*<br>FNTN Archive name*<br>FNTS Posizition*<br>FNTI Identification code*
**AD DATA ACCESS**
ADS DATA ACCESS SPECIFICATIONS*<br>ADSP Access profile*<br>ADSM Motivation*
**CM COMPILATION**
CMP COMPILATION*<br>CMPD Date*<br>CMPN Name*<br>RSR SCIENTIFIC REFERENCE<br>FUR IN CHARGE OFFICIAL*

Table S3. Number of specimens per chondrichthyan code at the "P. Doderlein" museum collection in the 1880, 1890, 1922 and 2024 inventories. – not found.

CODE	PRESENT IN 1880	ACQUIRED 1880-1890	PRESENT IN 1922	PRESENT IN 2024
118	2	-	1	1
119	18	-	18	16
120	37	-	32	29
121	53	-	46	41
123	1	-	1	1
129	23	-	23	-
138	2	-	2	2
139	36	-	36	33
143	27	-	27	25
147	45	-	45	42
148	3	-	3	5
151	169	-	185	37
152	96	-	96	1
153	30	-	30	1
154	9	-	9	1
158	6	-	3	1
119	-	1	-	-
435	-	1	1	-
439	-	1	1	1
478	-	1	1	1
486	-	1	1	1
487	-	1	-	-
488	-	2	2	2
489	-	1	1	1
494	-	1	1	1
500	-	1	1	1
501	-	1	1	1
502	-	1	1	1
551	-	1	1	1
564	-	3	-	-
565	-	2	2	2
567	-	1	1	1
589	-	1	1	-
594	-	1	1	-
595	-	1	-	1
600	-	1	1	1
601	-	1	1	1
602	-	1	1	1
638	-	1	-	-
658	-	1	1	1
693	-	1	-	-
694	-	1	1	1
695	-	1	1	-
709	-	1	1	1
710	-	2	-	-
715	-	1	1	1
716	-	1	1	1

741	-	1	1	1
742	-	1	1	1
743	-	1	1	1
744	-	1	1	-
762	-	1	1	1
763	-	1	1	1
765	-	1	1	-
803	-	2	2	2
804	-	1	1	1
805	-	1	1	-
807	-	1	1	-
811	-	1	1	1
847	-	1	1	1
852	-	1	1	-
974	-	1	1	1
975	-	1	1	1
1062	-	1	-	-
1115	-	1	1	-
1120	-	1	-	1
1270	-	1	1	1
440-441	-	1	1	-
737bis	-	1	1	1
2542 bis	-	-	1	1
2543 bis	-	-	1	1
2544 bis	-	-	1	1
2545 bis	-	-	2	2
2590 bis	-	-	1	1
2648 bis	-	-	4	6
2650 bis	-	-	25	29

Table S4. Updated species assignment based on morphological and genetic identification of specimens present in 2024. (* specimen with uncertain morphological species identification; ** indicates the impossibility to access to the specimen; - failed)

A	Code	Label name	Morphological identification	Genetic identification
AN	1	*Raja asterias*	*Raja asterias*	
AN	2	*Raja miraletus*	*Raja miraletus*	
AN	3	*Raja miraletus*	*Raja miraletus*	
AN	4	*Raja asterias*	*Raja asterias*	
AN	5	*Raja asterias*	*Raja asterias*	
AN	6	*Raja miraletus*	*Raja miraletus*	
AN	7	*Leucoraja circularis*	*Rostroraja alba*	
AN	8	*Leucoraja circularis*	*Leucoraja circularis*	
AN	9	*Leucoraja naevus*	*Leucoraja naevus*	
AN	10	*Leucoraja naevus*	*Leucoraja naevus*	
AN	11	*Raja asterias*	*Raja asterias*	
AN	12	*Rostroraja alba*	*Rostroraja alba*	
AN	13	*Leucoraja circularis*	*Leucoraja circularis*	

AN	14	*Rhinobatos rhinobatos*	*Rhinobatos rhinobatos*	
AN	15	*Rhinobatos rhinobatos*	*Rhinobatos rhinobatos*	
AN	16	*Myliobatis aquila*	*Myliobatis aquila*	
AN	17	*Aetomylaeus bovinus*	*Aetomylaeus bovinus*	
AN	18	*Torpedo marmorata*	*Torpedo marmorata*	
AN	19	*Tetronarce nobiliana*	*Tetronarce nobiliana*	
AN	20	*Raja radula*	*Raja radula*	
AN	21	*Raja radula*	*Raja radula*	
AN	22	*Raja radula*	*Raja radula*	
AN	23	*Raja radula*	*Raja radula*	
AN	24	*Dasyatis pastinaca*	*Dasyatis pastinaca*	
AN	25	*Pteroplatytrygon violacea*	*Pteroplatytrygon violacea*	
AN	26	*Mobula mobular*	*Mobula mobular*	
AN	27	*Etmopterus spinax*	*Etmopterus spinax*	
AN	28	*Heptranchias perlo*	*Heptranchias perlo*	
AN	29	*Raja clavata*	*Raja clavata*	
AN	30	*Raja clavata*	*Raja clavata*	
AN	31	*Squatina squatina*	*Squatina squatina**	-
AN	32	*Squatina squatina*	*Squatina squatina **	-
AN	33	*Squatina oculata*	*Squatina oculata**	-
AN	34	*Rostroraja alba*	*Rostroraja alba*	
AN	35	*Rostroraja alba*	*Raja brachyura*	
AN	36	*Gymnura altavela*	*Gymnura altavela*	
AN	37	*Aetomylaeus bovinus*	*Aetomylaeus bovinus*	
AN	38	*Carcharias taurus*	*Carcharias taurus*	
AN	39	*Dipturus batis*	*Dipturus batis**	*Dipturus batis*
AN	40	*Mustelus mustelus*	*Mustelus asterias*	
AN	41	*Squalus acanthias*	*Squalus blainville*	
AN	42	*Centrophorus granulosus*	*Centrophorus uyato*	
AN	43	*Scyliorhinus stellaris*	*Scyliorhinus stellaris*	
AN	44	*Dalatias licha*	*Dalatias licha*	
AN	45	*Chimaera monstrosa*	*Chimaera monstrosa*	
AN	46	*Oxynotus centrina*	*Oxynotus centrina*	
AN	47	*Chimaera monstrosa*	*Chimaera monstrosa*	
AN	48	*Sphyrna zygaena*	*Sphyrna zygaena*	
AN	49	*Heptranchias perlo*	*Heptranchias perlo*	
AN	50	*Echinorhinus brucus*	*Echinorhinus brucus*	
AN	51	*Raja asterias*	*Raja brachyura*	
AN	52	*Alopias vulpinus*	*Alopias vulpinus*	
AN	53	*Alopias vulpinus*	*Alopias vulpinus*	
AN	54	*Prionace glauca*	*Prionace glauca*	
AN	55	*Isurus oxyrinchus*	*Isurus oxyrinchus*	
AN	56	*Rostroraja alba*	*Rostroraja alba*	
AN	57	*Odontaspis ferox*	*Odontaspis ferox*	
AN	58	*Raja radula*	*Raja radula*	
AN	59	*Mustelus asterias*	*Mustelus mustelus*	

AN	60	*Carcharias taurus*	*Carcharias taurus*	
AN	61	*Dalatias licha*	*Dalatias licha*	
AN	62	*Bathytoshia centroura*	*Bathytoshia centroura*	
AN	63	*Isurus oxyrinchus*	*Isurus oxyrinchus*	
AN	64	*Isurus oxyrinchus*	*Isurus oxyrinchus*	
AN	65	*Isurus oxyrinchus*	*Isurus oxyrinchus*	
AN	66	*Odontaspis ferox*	*Odontaspis ferox*	
AN	67	*Dipturus oxyrinchus*	*Dipturus oxyrinchus*	
AN	68	*Carcharias taurus*	*Carcharias taurus*	
AN	69	*Raja asterias*	*Raja brachyura*	
AN	70	*Odontaspis ferox*	*Odontaspis ferox*	
AN	71	*Dasyatis pastinaca*	*Dasyatis pastinaca*	
AN	72	*Alopias vulpinus*	*Alopias vulpinus*	
AN	73	*Hexanchus griseus*	*Hexanchus griseus*	
AN	74	*Rostroraja alba*	*Rostroraja alba*	
AN	75	*Scyliorhinus stellaris*	*Scyliorhinus canicula*	
AN	76	*Aetomylaeus bovinus*	*Dasyatis centroura*	
AN	77	*Isurus oxyrinchus*	*Isurus oxyrinchus*	
AN	78	*Bathytoshia centroura*	*Bathytoshia centroura*	
AN	79	*Alopias vulpinus*	*Alopias vulpinus*	
AN	80	*Carcharhinus brachyurus*	*Carcharhinus brachyurus*	
AN	81	*Dipturus batis*	-	-
AN	82	*Aetomylaeus bovinus*	*Aetomylaeus bovinus*	
AN	83	*Squatina squatina*	*Squatina aculeata**	*Squatina aculeata*
AN	84	*Squatina squatina*	*Squatina oculata**	-
AN	85	*Odontaspis ferox*	*Odontaspis ferox*	
AN	86	*Odontaspis ferox*	*Odontaspis ferox*	
AN	87	*Rostroraja alba*	*Rostroraja alba*	
AN	88	*Myliobatis aquila*	*Myliobatis aquila*	
AN	89	*Hexanchus griseus*	*Hexanchus griseus*	
AN	90	*Isurus oxyrinchus*	*Isurus oxyrinchus*	
AN	91	*Sphyrna zygaena*	*Sphyrna zygaena*	
AN	92	*Rostroraja alba*	*Rostroraja alba*	
AN	93	*Prionace glauca***	-	
AN	94	*Carcharias taurus*	*Odontaspis ferox*	
AN	95	*Echinorhinus brucus*	*Echinorhinus brucus*	
AN	96	*Raja radula*	*Raja radula*	
AN	97	*Sphyrna zygaena*	*Sphyrna zygaena*	
AN	98	*Hexanchus griseus*	*Hexanchus griseus*	
AN	99	*Raja clavata*	*Raja clavata*	
AN	100	*Prionace glauca*	*Prionace glauca*	
AN	101	*Isurus oxyrinchus*	*Isurus oxyrinchus*	
AN	102	*Mobula mobular*	*Mobula mobular*	
AN	103	*Raja undulata*	*Raja clavata*	
AN	104	*Dipturus oxyrinchus*	*Dipturus oxyrinchus*	
AN	105	*Prionace glauca*	*Carcharhinus plumbeus*	

AN	106	*Hexanchus griseus*	*Hexanchus griseus*	
AN	107	*Dasyatis pastinaca*	*Dasyatis pastinaca*	
AN	108	*Carcharodon carcharias*	*Carcharodon carcharias*	
AN	109	*Prionace glauca*	*Carcharhinus plumbeus*	
AN	110	*Prionace glauca*	*Echinorhinus brucus*	
AN	111	*Prionace glauca*	*Carcharhinus plumbeus*	
AN	112	*Lamna nasus*	*Lamna nasus*	
AN	113	*Odontaspis ferox*	*Odontaspis ferox*	
AN	114	*Aetomylaeus bovinus*	*Aetomylaeus bovinus*	
AN	115	*Carcharodon carcharias*	*Carcharodon carcharias*	
AN	116	*Bathytoshia centroura*	*Bathytoshia centroura*	
AN	117	*Prionace glauca*	*Carcharhinus plumbeus*	
AN	118	*Prionace glauca*	*Prionace glauca*	
AN	119	*Centrophorus granulosus*	*Centrophorus uyato*	
AN	120	*Raja clavata*	*Raja clavata*	
AN	121	*Leucoraja circularis*	-	-
AN	122	*Centrophorus granulosus*	*Centrophorus uyato*	
AN	123	*Prionace glauca*	*Carcharhinus plumbeus*	
AN	124	*Galeorhinus galeus*	*Galeorhinus galeus*	
AN	125	*Dalatias licha*	*Dalatias licha*	
AN	127	*Bathytoshia centroura*	*Bathytoshia centroura*	
AN	128	*Carcharodon carcharias*	*Carcharodon carcharias*	
AN	129	*Centrophorus granulosus*	*Centrophorus uyato*	
AN	130	*Centrophorus granulosus*	*Centrophorus granulosus*	
AN	131	*Prionace glauca*	*Carcharhinus plumbeus*	
AN	132	*Odontaspis ferox*	*Carcharias taurus*	
AN	133	*Sphyrna zygaena*	*Sphyrna zygaena*	
AN	134	*Galeorhinus galeus*	*Galeorhinus galeus*	
AN	135	*Scyliorhinus canicula*	*Scyliorhinus stellaris*	
AN	136	*Aetomylaeus bovinus*	*Aetomylaeus bovinus*	
AN	137	*Pteroplatytrygon violacea*	*Pteroplatytrygon violacea*	
AN	138	*Bathytoshia centroura*	*Bathytoshia centroura*	
AN	139	*Galeorhinus galeus*	*Galeorhinus galeus*	
AN	140	*Squatina squatina*	*Squatina aculeata**	*Squatina aculeata*
AN	141	*Isurus oxyrinchus*	*Isurus oxyrinchus*	
AN	142	*Squatina oculata*	*Squatina aculeata**	-
AN	143	*Echinorhinus brucus*	*Echinorhinus brucus*	
AN	144	*Prionace glauca*	*Carcharhinus plumbeus*	
AN	145	*Carcharodon carcharias*	*Carcharodon carcharias*	
AN	149	*Rostroraja alba*	*Rostroraja alba*	
AN	182	*Pristis pristis*	*Anoxypristis cuspidata*	*Anoxypristis cuspidata* [80]
AN	183	*Pristis pristis*	*Pristis zijsron*	*Pristis zijsron* [80]
AN	184	*Pristis pristis*	*Pristis pectinata*	*Pristis pectinata* [80]
AN	185	*Pristis pristis*	*Pristis pectinata*	*Pristis pectinata* [80]
AN	186	*Pristis pristis*	*Pristis zijsron*	*Pristis zijsron* [80]
AN	359	*Carcharhinus brachyurus*	*Carcharhinus brachyurus*	

AN	405	*Squatina oculata*	*Squatina aculeata**	*Squatina aculeata*
AN	406	*Leucoraja circularis*	*Rostroraja alba**	*Rostroraja alba*
AN	664	*Dipturus oxyrinchus*	*Dipturus oxyrinchus*	
AN	1234	*Isurus oxyrinchus*	*Isurus oxyrinchus*	
AN	1235	*Isurus oxyrinchus*	*Prionace glauca*	
AN	1236	*Isurus oxyrinchus*	*Odontaspis ferox*	
AN	1238	*Mustelus asterias*	*Mustelus asterias*	
AN	1330	*Anoxypristis cuspidata*	*Anoxypristis cuspidata*	
AN	1331	*Heterodontus zebra*	*Heterodontus zebra*	
AN	1410	*Cetorhinus maximus*	*Cetorhinus maximus*	
P	515	*Oxynotus centrina*	*Oxynotus centrina*	
P	516	*Oxynotus centrina*	*Oxynotus centrina*	
P	517	*Echinorhinus brucus*	*Echinorhinus brucus*	
P	518	*Odontaspis ferox*	*Carcharias taurus*	
P	519	*Dalatias licha*	*Dalatias licha*	
P	520	*Mustelus asterias*	*Mustelus mustelus*	
P	521	*Dalatias licha*	*Dalatias licha*	
P	522	*Carcharias taurus*	*Carcharias taurus*	
P	523	*Odontaspis ferox*	*Odontaspis ferox*	
P	524	*Hexanchus griseus*	*Hexanchus griseus*	
P	525	*Etmopterus spinax*	*Etmopterus spinax*	
P	526	*Etmopterus spinax*	*Etmopterus spinax*	
P	527	*Galeorhinus galeus*	*Galeorhinus galeus*	
P	528	*Somniosus rostratus*	*Somniosus rostratus*	
P	529	*Dalatias licha*	*Dalatias licha*	
P	530	*Dalatias licha*	*Dalatias licha*	
P	531	*Galeorhinus galeus*	*Galeorhinus galeus*	
P	532	*Centrophorus granulosus*	*Centrophorus uyato*	
P	533	*Mustelus mustelus*	*Mustelus mustelus*	
P	534	*Mustelus asterias*	*Mustelus asterias*	
P	535	*Prionace glauca*	*Prionace glauca*	
P	536	*Scyliorhinus canicula*	*Scyliorhinus canicula*	
P	537	*Prionace glauca*	*Prionace glauca*	
P	538	*Centrophorus granulosus*	*Centrophorus uyato*	
P	539	*Heptranchias perlo*	*Heptranchias perlo*	
P	540	*Heptranchias perlo*	*Heptranchias perlo*	
P	541	*Heptranchias perlo*	*Heptranchias perlo*	
P	542	*Scyliorhinus stellaris*	*Scyliorhinus canicula*	
P	543	*Scyliorhinus stellaris*	*Scyliorhinus canicula*	
P	544	*Scyliorhinus stellaris*	*Scyliorhinus canicula*	
P	547	*Isurus oxyrinchus*	*Isurus oxyrinchus*	
P	548	*Isurus oxyrinchus*	*Isurus oxyrinchus*	
P	549	*Odontaspis ferox*	*Odontaspis ferox*	
P	550	*Chimaera monstrosa*	*Chimaera monstrosa*	
P	551	*Carcharhinus plumbeus*	*Carcharhinus plumbeus*	
P	552	*Alopias vulpinus*	*Alopias vulpinus*	

P	553	*Alopias vulpinus*	*Alopias vulpinus*
P	554	*Carcharhinus plumbeus*	*Carcharhinus plumbeus*
P	555	*Chimaera monstrosa*	*Chimaera monstrosa*
P	556	*Sphyrna zygaena*	*Sphyrna zygaena*
P	557	*Sphyrna zygaena*	*Sphyrna zygaena*
P	558	*Sphyrna zygaena*	*Sphyrna zygaena*
P	559	*Sphyrna zygaena*	*Sphyrna zygaena*
P	560	*Rhinobatos rhinobatos*	*Rhinobatos rhinobatos*
P	561	*Squatina aculeata*	*Squatina aculeata*
P	562	*Rhinobatos rhinobatos*	*Rhinobatos cemiculus*
P	563	*Squatina oculata*	*Squatina aculeata*
P	564	*Squatina oculata*	*Squatina oculata*
P	565	*Leucoraja naevus*	*Leucoraja naevus*
P	566	*Leucoraja circularis*	*Leucoraja circularis*
P	567	*Raja miraletus*	*Raja miraletus*
P	568	*Dipturus oxyrinchus*	*Dipturus oxyrinchus*
P	569	*Leucoraja circularis*	*Leucoraja circularis*
P	570	*Raja asterias*	*Raja asterias*
P	571	*Gymnura altavela*	*Gymnura altavela*
P	572	*Dipturus oxyrinchus*	*Dipturus oxyrinchus*
P	573	*Gymnura altavela*	*Gymnura altavela*
P	574	*Rostroraja alba*	*Rostroraja alba*
P	575	*Torpedo torpedo*	*Torpedo torpedo*
P	576	*Bathytoshia centroura*	*Bathytoshia centroura*
P	577	*Leucoraja fullonica*	*Leucoraja fullonica*
P	578	*Torpedo marmorata*	*Torpedo marmorata*
P	579	*Dasyatis pastinaca*	*Dasyatis pastinaca*
P	581	*Tetronarce nobiliana*	*Tetronarce nobiliana*
P	582	*Aetomylaeus bovinus*	*Aetomylaeus bovinus*
P	583	*Rostroraja alba*	*Rostroraja alba*
P	584	*Chimaera monstrosa*	*Chimaera monstrosa*
P	585	*Glaucostegus cemiculus*	*Glaucostegus cemiculus*
P	586	*Raja brachyura*	*Raja polystigma*
P	587	*Torpedo torpedo*	*Torpedo torpedo*
P	588	*Rostroraja alba*	*Rostroraja alba*
P	589	*Rhinobatos rhinobatos*	*Rhinobatos rhinobatos*
P	590	*Rhinobatos rhinobatos*	*Rhinobatos rhinobatos*
P	591	*Rhinobatos rhinobatos*	*Rhinobatos rhinobatos*
P	592	*Rostroraja alba*	*Rostroraja alba*
P	593	*Raja brachyura*	*Raja brachyura*
P	594	*Raja clavata*	*Raja clavata*
P	595	*Gymnura altavela*	*Gymnura altavela*
P	596	*Aetomylaeus bovinus*	*Aetomylaeus bovinus*
P	597	*Raja radula*	*Raja radula*
P	598	*Myliobatis aquila*	*Myliobatis aquila*
P	599	*Pteroplatytrygon violacea*	*Pteroplatytrygon violacea*

P	600	*Bathytoshia centroura*	*Bathytoshia centroura*	
P	601	*Myliobatis aquila*	*Myliobatis aquila*	
P	602	*Raja brachyura*	*Raja polystigma*	
P	603	*Torpedo torpedo*	*Torpedo torpedo*	
P	604	*Raja brachyura*	*Raja polystigma*	
P	605	*Leucoraja circularis*	*Leucoraja circularis*	
P	606	*Raja montagui*	-	*Raja radula*
P	607	*Raja asterias*	*Raja asterias*	
P	608	*Raja radula*	*Raja radula*	
P	609	*Leucoraja circularis*	*Leucoraja circularis*	
P	610	*Raja asterias*	*Raja asterias*	
P	611	*Raja clavata*	*Raja clavata*	
P	612	*Raja asterias*	*Raja polystigma*	
P	613	*Raja miraletus*	*Raja miraletus*	
P	614	*Dasyatis pastinaca*	*Dasyatis pastinaca*	
P	615	*Raja miraletus*	*Raja miraletus*	
P	616	*Raja montagui*	-	*Raja radula*
P	617	*Raja clavata*	*Raja clavata*	
P	618	*Raja asterias*	*Raja brachyura*	
P	619	*Raja radula*	*Raja radula*	
P	620	*Raja clavata*	*Raja clavata*	
P	621	*Dipturus oxyrinchus*	*Dipturus oxyrinchus*	
P	622	*Raja asterias*	*Raja polystigma*	
P	623	*Raja clavata*	*Raja clavata*	
P	624	*Raja asterias*	*Raja asterias*	
P	625	*Raja radula*	*Raja radula*	
P	626	*Torpedo marmorata*	*Torpedo marmorata*	
P	627	*Leucoraja circularis*	*Leucoraja circularis*	
P	628	*Raja brachyura*	-	-
P	629	*Torpedo marmorata*	*Torpedo marmorata*	
P	630	*Mobula mobular*	*Mobula mobular*	
P	631	*Squalus blainville*	*Squalus blainville*	
P	632	*Centrophorus granulosus*	*Centrophorus uyato*	
P	633	*Squalus blainville*	*Squalus blainville*	
P	634	*Centrophorus uyato*	*Centrophorus uyato*	
P	645	*Raja polystigma*	*Raja brachyura*	
P	649	*Rhinobatos rhinobatos*	*Rhinobatos cemiculus*	
P	650	*Rhinobatos rhinobatos*	*Rhinobatos cemiculus*	
P	651	*Dipturus nidarosiensis*	*Dipturus nidarosiensis**	*Dipturus nidarosiensis*
P	652	*Rostroraja alba*	*Rostroraja alba*	
P	653	*Dipturus intermedius*	*Dipturus batis*	
P	654	*Dasyatis pastinaca*	*Dasyatis pastinaca*	
P	657	*Cetorhinus maximus*	*Cetorhinus maximus*	
P	658	*Squatina squatina*	*Squatina squatina*	
P	660	*Dipturus oxyrinchus*	*Dipturus batis*	

Table S5. DNA concentration, sequence length, percentage of identity and species identification for 16 barcoded samples.

Museum ID	DNA concentration (ng/μL)	% of identity NCBI/BOLD	Species	GenBank Accession Numbers
AN 31	3.1	-	-	-
AN 32	3.2	-	-	-
AN 33	1.8	-	-	-
AN 39	29.4	100/na	*Dipturus batis*	PX070301
AN 81	3.5	nd	nd	nd
AN 83	1.3	100/na	*Squatina aculeata*	PX070299
AN 84	4.0	nd	nd	nd
AN121	26.2	nd	nd	nd
AN 140	8.0	99.53/na	*Squatina aculeata*	PX070297
AN 142	3.9	-	-	-
AN 405	3.2	100/na	*Squatina aculeata*	PX070298
AN 406	22.8	99.06/na	*Rostroraja alba*	PX070300
P606	2.8	100/na	*Raja polystigma/ Raja radula*	PX070302
P616	18.1	100/na	*Raja polystigma/ Raja radula*	PX070303
P628	19.3	nd	nd	nd
P651	21.0	100/na	*Dipturus nidarosiensis*	PX070304

nd: not determined; na: not available; -: failed
